# Regulation of the perilymphatic–endolymphatic water shunt in the cochlea by membrane translocation of aquaporin-5

**DOI:** 10.1007/s00424-015-1720-6

**Published:** 2015-07-25

**Authors:** A. Eckhard, A. Dos Santos, W. Liu, M. Bassiouni, H. Arnold, C. Gleiser, B. Hirt, C. Harteneck, M. Müller, H. Rask-Andersen, H. Löwenheim

**Affiliations:** Department of Otorhinolaryngology—Head & Neck Surgery, University of Tübingen Medical Centre, Tübingen, Germany; Department of Surgical Sciences, Section of Otolaryngology, Uppsala University Hospital, Uppsala, Sweden; Department of Pharmacology and Experimental Therapy, Institute of Experimental and Clinical Pharmacology and Toxicology, Interfaculty Center of Pharmacogenomics and Pharmaceutical Research (ICePhA), University of Tübingen, Tübingen, Germany; Institute of Anatomy, University of Tübingen, Tübingen, Germany; Department of Otolaryngology, Massachusetts Eye and Ear Infirmary, Harvard Medical School, Boston, MA USA; School of Medicine and Health Sciences - European Medical School, University Hospital of Otorhinolaryngology, Medical Campus University of Oldenburg, Steinweg 13-17, 26122 Oldenburg, Germany; Research Center of Neurosensory Science, University of Oldenburg, 26111 Oldenburg, Germany; Cluster of Excellence Hearing4all, University of Oldenburg, 26111 Oldenburg, Germany

**Keywords:** Aquaporin, Cochlea, Water permeability, Muscarinic, Pilocarpine, Ménière’s disease

## Abstract

Volume homeostasis of the cochlear endolymph depends on radial and longitudinal endolymph movements (LEMs). LEMs measured in vivo have been exclusively recognized under physiologically challenging conditions, such as experimentally induced alterations of perilymph osmolarity or endolymph volume. The regulatory mechanisms that adjust LEMs to the physiological requirements of endolymph volume homeostasis remain unknown. Here, we describe the formation of an aquaporin (AQP)-based “water shunt” during the postnatal development of the mouse cochlea and its regulation by different triggers. The final complementary expression pattern of AQP5 (apical membrane) and AQP4 (basolateral membrane) in outer sulcus cells (OSCs) of the cochlear apex is acquired at the onset of hearing function (postnatal day (p)8–p12). In vitro, hyperosmolar perfusion of the perilymphatic fluid spaces or the administration of the muscarinic agonist pilocarpine in cochlear explants (p14) induced the translocation of AQP5 channel proteins into the apical membranes of OSCs. AQP5 membrane translocation was blocked by the muscarinic antagonist atropine. The muscarinic M3 acetylcholine (ACh) receptor (M3R) was identified in murine OSCs via mRNA expression, immunolabeling, and in vitro binding studies using an M3R-specific fluorescent ligand. Finally, the water shunt elements AQP4, AQP5, and M3R were also demonstrated in OSCs of the human cochlea. The regulation of the AQP4/AQP5 water shunt in OSCs of the cochlear apex provides a molecular basis for regulated endolymphatic volume homeostasis. Moreover, its dysregulation or disruption may have pathophysiologic implications for clinical conditions related to endolymphatic hydrops, such as Ménière’s disease.

**Electronic supplementary material:**

The online version of this article (doi:10.1007/s00424-015-1720-6) contains supplementary material, which is available to authorized users.

## Introduction

Volume regulation of the cochlear fluids, including the endolymph and perilymph, is crucial for cochlear function and the transduction of sound. Based on electric current measurements [91] and the dispersal of tracer substances [2, 4, 22, 23, 45, 72–74] or ionic volume markers [76], two main homeostatic mechanisms of inner ear fluid regulation have been proposed, i.e., “radial endolymph movements” (REMs) [67] and “longitudinal endolymph movements” (LEMs) [27]. REMs occur locally across the cochlear duct epithelium by endolymphatic ion secretion across the stria vascularis and endolymphatic ion resorption by the sensory hair cells within the organ of Corti [91] and other non-sensory epithelial cells within the cochlear duct [58, 62]. REMs presumably constitute the predominant homeostatic mechanism under normal conditions. LEMs, in contrast, have been measured exclusively under experimentally induced pathophysiologic conditions, such as disturbed perilymph osmolarity [76] or endolymph volume [75]. In particular, LEMs within the scala media were directed apically (baso-apical fluid flow) when the perilymph osmolarity in the scalae vestibuli and tympani was increased (400 mOsm/L [76]) or when the endolymph volume in the scala media was reduced [75]. LEMs in the scala media were directed basally (apico-basal fluid flow) when the osmolarity of the perilymph was decreased compared with that of the endolymph [76] or when the volume of the endolymph was increased by artificial endolymph injections into the scala media [75]. Thus, bidirectional LEMs may correct endolymph volume disturbances or osmotic imbalances under physiologically challenging conditions when REMs are insufficient to maintain endolymph homeostasis [75, 76]. The molecular mechanisms in the inner ear epithelium that trigger and regulate LEMs are, however, largely unknown. These molecular mechanisms are of particular pathophysiological interest because their disturbances and the consecutive stagnation of LEMs have been implicated in the generation of endolymphatic hydrops in Ménière’s disease [27, 52].

We previously described a “water shunt” between the perilymphatic and endolymphatic fluid spaces in the cochlear apex of various mammalian species, including rats, mice, gerbils [33], guinea pigs [21, 33], and humans [reviewed in 19, 20, 33]. This water shunt is established by the membranous localization of two molecular water channel (aquaporin, AQP) subtypes in the basolateral (AQP4) and apical (AQP5) membrane domains of outer sulcus cells (OSCs), which reside at the perilymph–endolymph barrier. We recently determined the transcellular water permeability of these AQP4/5-expressing OSCs in response to perilymphatic osmolarity shifts (osmotic water permeability coefficient, P_f_) at 156.90 × 10^−3^ cm s^−1^ in the guinea pig cochlea [21]. This high osmotic water permeability is more than 255-fold that of other epithelial cells at the cochlear perilymph–endolymph barrier (P_f_ = 6.15 × 10^−4^ cm s^−1^ [21]) and is comparable to that of epithelia, which highly depend on AQP-facilitated water transport, e.g., certain segments of the kidney tubule epithelium. We proposed that under osmotically challenging conditions induced in vivo [76], bulk water flow across the AQP4/5-expressing OSCs in the cochlear apex putatively induces LEMs in the baso-apical or apico-basal direction [21]. The AQP–water shunt thereby potentially facilitates the rehydration or dehydration of the endolymphatic fluid compartment and contributes to the maintenance of endolymphatic volume homeostasis.

Notably, most epithelia that accomplish AQP-facilitated fluid homeostasis adjust their rates of transepithelial water flow to the prevailing physiological conditions by controlling the membrane density of AQP channel proteins. Short-term regulation of the AQP membrane density is commonly achieved via the translocation of pre-formed AQP channel proteins that reside in cytoplasmic membrane vesicles, preferentially in the apical plasma membrane. Various translocation triggers in different cell types have been described for eight of the 12 mammalian AQP subtypes (AQP1–5 and AQP7–9) (reviewed in [16]).

Based on the previously established polarized membrane localization of AQP4 (basolateral) and AQP5 (apical) in cochlear OSCs and the various membrane translocation mechanisms for AQP5 that have been described in other epithelia, we hypothesize in the present study that water flow through the cochlear AQP–water shunt can be adapted to the prevailing homeostatic requirements by regulated apical membrane trafficking of AQP5 channel proteins in cochlear OSCs. Therefore, we investigated whether two trigger pathways for the membrane translocation of AQP5 that have already been established in other epithelia (extracellular osmolarity changes [34] and autonomic (muscarinic) activity [41]) are also present in cochlear OSCs. In a first step, we performed immunofluorescence double-labeling of AQP4 and AQP5 in the murine cochlea during postnatal development to reveal the onset of co-localized AQP4 and AQP5 expression in OSCs (i.e., the formation of the perilymphatic–endolymphatic water shunt). We then performed immunofluorescence labeling and in vitro ligand binding assays to demonstrate the localization of muscarinic (M3) acetylcholine (ACh) receptors in OSCs in the mouse (p14) cochlea. Finally, explants of the murine (p14) cochlear duct were used to investigate in vitro the effect of known AQP5 translocation triggers, i.e., extracellular osmolarity changes and autonomic (parasympathetic) activity [41], on the subcellular localization of AQP5 in cochlear OSCs.

## Materials and methods

### Animals

Animal use for organ explantation was approved by the Committee for Animal Experiments of the Regional Council (Regierungspräsidium) of Tübingen (dated February 4, 2013). The animals were maintained in an in-house animal facility with free access to food and water under a standard 12-h light/dark cycle. NMRI mice at postnatal days (p) 0, 2, 4, 8, 10, 12, 14, 16, 32, 64, and 128 were obtained from an in-house breeding colony. The cochleae from three mice at each developmental time point from p0 to p128 and from 8 mice at p14 were used to determine the immunolocalization of AQP4 and AQP5 in OSCs during postnatal development, as well as the mRNA expression and the immunolocalization of M3R at p14. The cochleae from two mice at p4 and p38 were processed for Epon embedding. The cochleae from 23 mice at p14–p16 were used for the in vitro M3R ligand binding assay and to determine the translocation of AQP5 in OSCs after in vitro incubation in low-osmolarity or high-osmolarity solutions or with muscarinic antagonist/agonists, respectively. Positive control tissue from the parotid glands was obtained from the same mice from which the cochleae were removed.

### Inner ear dissection

Animals at postnatal ages p0–p4 were decapitated using sharp scissors. Older animals (p8–p128) were suffocated with carbon dioxide (CO_2_) prior to decapitation. The dissection of the bony labyrinth of the inner ear was conducted according to previously described methods [33] in HEPES-buffered Hank’s solution (HHBSS; 5.36 mM KCl, 0.41 mM MgSO_4_·7H_2_O, 0.49 mM MgCl_2_·6H_2_O, 141.5 mM NaCl, 9.99 mM C_8_H_18_N_2_O_4_S (HEPES), 3.42 mM C_5_H_10_N_2_O_3_ (l-glutamine), 1.57 mM CaCl_2_·2H_2_O, 6.31 mM d-glucose monohydrate, aqua dest.; osmolality: 285 mOsm/L, pH: 7.32 as measured in one batch; the pharmacy of the University Hospital of Tübingen).

### In vitro perilymphatic perfusion and incubation of cochlear explants

For in vitro perilymphatic perfusion of explanted cochleae with HHBSS (285 mOsm/L), low-osmolarity solution (HHBSS diluted with aqua dest., 200 mOsm/L), or high-osmolarity solution (HHBSS supplemented with sucrose, 400 mOsm/L), the bony capsules of the cochleae were punctured in the helicotrema region to create an outlet for the perfusion solutions; otherwise, the cochlear specimens remained intact. Approximately, 300 μL of the respective solutions was pre-warmed (37 °C) and slowly injected through the round and oval windows into the scala tympani and the scala vestibuli, respectively, using a syringe with a 30-gauge needle (Microlance 3, BD Biosciences, San Jose, CA, USA). The perfused cochleae were subsequently immersed in the solution that was used for the perfusion of the respective specimen and incubated at 37 °C for 10 min.

From the cochleae, which were utilized for in vitro incubation with M3R antagonist/agonists, the entire apical part of the bony capsule was removed to expose the apical turn of the cochlear duct. The specimens were immersed in pre-warmed HHBSS supplemented with either the M3R agonist pilocarpine hydrochloride (10 μM; Sigma-Aldrich, St. Louis, MO, USA) only or with pilocarpine and the M3R antagonist atropine (100 μM; Sigma-Aldrich) and incubated at 37 °C for 10 min. Three cochleae were perfused with a hyperosmolar solution (400 mOsm/L) supplemented with atropine (100 μM) and were subsequently incubated for 10 min in the respective perfusion solution. Positive control tissue from the parotid glands of the same animals was sliced to a thickness of approximately 500 μm using a razor blade and processed in parallel.

All cochlear and parotid gland specimens were fixed after in vitro incubation in ice-cold 4 % paraformaldehyde (4 % PFA, Carl Roth GmbH, Karlsruhe, Germany) for 1 h. Three cochleae (*n* = 3) derived from three different animals were assigned to each of the six experimental groups (Supplementary Table 1).

### In vitro mapping of muscarinic M3 receptors using a receptor-specific fluorescent ligand

For in vitro M3R ligand binding experiments, the bony cochlear capsule was removed in the apical region of the cochlea, and the spiral ligament in the apical cochlear turn was dissected. Spiral ligament specimens were incubated at 37 °C for 20 min with the fluorescent-labeled pirenzepine derivative M3–633–AN (100 nM in 0.1 % dimethyl sulfoxide (DMSO), Abcam, Cambridge, MA, USA) after 10 min of pre-incubation in 0.1 % DMSO. According to the manufacturer’s information, the receptor selectivity of M3–633–AN is highest for M3R (pK_D_ = 7.97) compared with other muscarinic receptor subtypes (M1: pK_D_ = 6.24, M5: pK_D_ = 6.29). In the negative control experiments, the specimens were either incubated for 30 min in 0.1 % DMSO or pre-incubated for 10 min with the unlabeled M3R competitor 4-diphenylacetoxy-*N*-methyl-piperidine methiodide (4-DAMP, 10 μM, Sigma-Aldrich; M3: pK_B_ = 8.83 [26]) in 0.1 % DMSO prior to 20 min of incubation with M3–633–AN. After in vitro incubation, the specimens were subsequently fixed in ice-cold 4 % PFA for 20 min labeled with Alexa Fluor® 568 phalloidin (dilution 1:400; Molecular Probes–Invitrogen, Carlsbad, CA, USA) and counterstained using 4′,6-diamidino-2-phenylindol (DAPI, Molecular Probes–Invitrogen, dilution 1:100 in PBS). All specimens were protected from light during and after the in vitro incubation. Whole-mount specimens from three cochleae (*n* = 3) derived from three independent animals were analyzed per experimental group (Supplementary Table 2).

### Cryoembedding, Epon embedding, and sectioning

After fixation, all tissue specimens were immersed for 1 h in phosphate-buffered saline (PBS) and subsequently decalcified overnight in 2 mM ethylene-diamine-tetra-acetic acid (EDTA). The specimens were embedded in Cryogel or in Epon according to previously described standard methods [33]. The cryosections were cut at 10 μm thickness using a cryostat (Leica CM3050, Leica Biosystems, Wetzlar, Germany) and were mounted on SuperFrost® Plus microscope slides (Langenbrinck, Emmendingen, Germany). The sections were cut at 10 μm thickness using a HM 355S rotary microtome (Thermo Scientific, Walldorf, Germany) and mounted in Permount on microscope slides after toluidine blue staining.

### Human tissues

The use of human specimens for this study was approved by the responsible ethics committees of the Uppsala University Hospital (no. 99398, no. 22/9 1999, no. C254/4, and no. C45/7 2007) and the University of Tübingen Medical Centre (no. 443/2013BO2). The human cochlea that was used in this study was provided by H. Rask–Andersen (Uppsala, Sweden). The specimen was obtained during an intracranial tumor removal surgery from a patient with petroclival meningioma. The removal of the inner ear was necessary for the radical removal of the life-threatening tumor. The patient had no history of otologic disease, and the pre-operatively measured pure tone audiograms (PTA) were within the normal range for the patient’s age; the cochlear specimen was therefore considered normal regarding its morphology and function. The human salivary gland specimen was obtained from a surgical sample of the parotid gland that had been removed during a tumor removal surgery at the Department of Otorhinolaryngology—Head and Neck Surgery of the University of Tübingen Medical Centre. The cochlea and the salivary gland specimens used in this study were derived from two different patients. The study was conducted according to the Declaration of Helsinki, and informed consent of the patients was obtained.

The processing of the human cochlea for immunohistochemical studies was performed as previously described [20, 33]. The human parotid gland specimen was processed as described for the murine salivary gland tissue.

### Real-time quantitative PCR

Tissue samples were dissected/isolated and immediately stored in RNA*later*® (Applied Biosystems, Darmstadt, Germany) for RNA isolation. Total RNA isolation and DNase I treatment were performed using a RNAqueous® Micro Kit (AM1931, Ambion, Austin, TX, USA), and complimentary DNA synthesis was performed with a Transcriptor High Fidelity cDNA Synthesis Kit (05081955001, Roche Diagnostics, Mannheim, Germany), both according to the manufacturer’s instructions. The transcript levels were determined using the fluorescence-based Quant-iT™ assays with a Qubit™ Quantitation Platform (Invitrogen). For each qPCR amplification reaction, the template was adjusted to 5 ng of total cDNA in a total volume of 20 μL, and the reaction was performed using LightCycler® 480 Probes Master Mix (04707494001, Roche Diagnostics) according to the manufacturer’s protocols. The probes were designed using RealTime ready Single Assays (Roche Applied Science; Assay IDs: *Chrm3* (311010), *Tbp* (300314), *Ubc* (311816), and *Actb* (300236)). Measurements were conducted in triplicates, and a no-template blank served as the negative control. C_T_ (threshold cycle) values were determined using the LightCycler® 480 Software release 1.5.0 SP4 (Roche Diagnostics). Assay-specific PCR efficiencies (E = 10^(−1/slope)^) and errors (mean squared error of the single data points fit to the regression line) were calculated via the software using the slope of standard curve experiments, which were conducted with mRNA from the murine organ of Corti (p4) tissue (reference genes) and the murine spinal cord (p14) tissue for *Chrm3* (Supplementary Figure 3). Transcription levels were normalized to the average of two to three housekeeping genes and then to the reference sample using the formula 2^−ΔΔC^_T_ according to [60] (Supplementary Table 3).

### Immunofluorescence labeling

Immunofluorescence labeling of M3R, the water channel proteins AQP4 and AQP5, E-cadherin, the inward rectifier-type potassium channel Kir4.1 (Kir4.1), and Flottilin-2 (Flot-2) was performed using the polyclonal antibodies anti-muscarinic acetylcholine receptor M3 antibody raised in rabbit (dilution 1:100; ab87199, Abcam), anti-AQP4 antibody raised in goat (1:100; sc-9888, Santa Cruz Biotechnology, Heidelberg, Germany), anti-AQP5 antibody raised in rabbit (1:200; AB3559 Merck-Millipore, Darmstadt, Germany), anti-Kir4.1 antibody raised in goat (1:100; sc-23637, Santa Cruz), anti-Flot-2 antibody raised in mouse (1: 50; sc-28320, Santa Cruz), and a monoclonal anti-Uvomorulin/E-cadherin antibody raised in rat (1:800; U3254, Sigma-Aldrich). The primary antibodies were visualized with an Alexa 488-conjugated anti-rabbit secondary antibody (dilution 1:400; Molecular Probes–Invitrogen), an Alexa 568-conjugated anti-mouse secondary antibody (dilution 1:400; Molecular Probes–Invitrogen), an Alexa 546-conjugated anti-goat secondary antibody (dilution 1:400; Molecular Probes–Invitrogen), or an Alexa 594-conjugated anti-rat secondary antibody (dilution 1:400; Molecular Probes–Invitrogen), all of which were raised in donkey. All antibodies were diluted in PBS supplemented with 0.1 % Triton X-100 and 0.5 % normal donkey serum (NDS). Membrane-associated filamentous actin (F-actin) was labeled using Alexa Fluor® 568 phalloidin (dilution 1:400; Molecular Probes–Invitrogen). For antigen retrieval prior to immunolabeling of M3R, the cryosections were immersed in 10 mM sodium citrate buffer (pH 6.0) and boiled in a steamer at 100 °C for 10 min. Immunolabeling of Kir4.1 was performed after antigen retrieval in 1 % sodium dodecyl sulfate (SDS) for 10 min. Following the antigen retrieval procedures, the slides were washed thoroughly in PBS and immunolabeled. For primary antibodies directed against M3R and AQP5, preabsorption experiments were performed by pre-incubating 0.1 μg/μL of the antibody with 0.2 μg/μL of the respective control peptide for 1 h prior to the immunolabeling procedures. The cryosections were immunolabeled in a humidified chamber, and lateral wall whole-mount preparations were labeled during free-floating incubation. The cryosections were counterstained using DAPI (Molecular Probes–Invitrogen, dilution 1:100 in PBS). All cryosections and lateral wall preparations were coverslipped using FluorSave™ mounting medium (Calbiochem–Merck, Darmstadt, Germany).

### Microscopic analysis

Immunolabeled whole-mount preparations and cryosections were analyzed using an Axioplan 2 epifluorescence microscope (Zeiss, Göttingen, Germany), an Axio Imager (M2 upright) with ApoTome.2 unit (Zeiss), or a Zeiss 510 laser-scanning microscope (Zeiss).

### Measurement of the baso-apical extent of AQP4 and AQP5 immunofluorescence in lateral wall whole-mount specimens

**Fig. 1:**
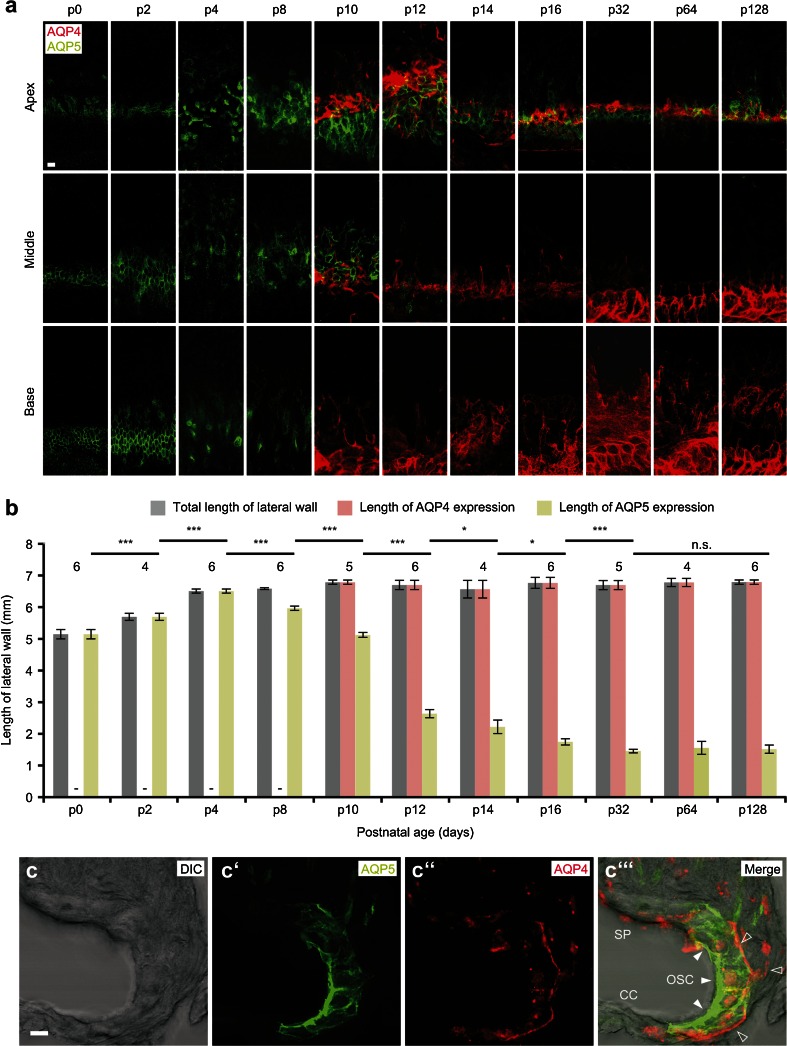
Expression of AQP4 and AQP5 in the outer sulcus cell (OSC) region of the mouse cochlear duct during postnatal development. **a** Immunofluorescence double labeling of AQP4 and AQP5 in spiral ligament whole-mount preparations from the mouse cochlea between postnatal days (p) 0 and p128. Representative confocal images of fluorescence signals were obtained for specimens from the apical (*first row*), middle (*second row*), and basal turn (*third row*) of the cochlea. **b** Measurements of the total baso-apical length of the spiral ligament (*gray bars*) and the baso-apical lengths of AQP4 (*red bars*), and AQP5 fluorescence (*green bars*) in the outer sulcus region. Length measurements were obtained on whole-mount specimens derived from 4 to 6 cochleae per developmental age (numbers above bars). (Error bars indicate SD, Student’s *t* test: *p ≤ 0.05; **p ≤ 0.01; ***p ≤ 0.001; *n.s.* not significant). **c–c”’** Confocal images of the subcellular localization of AQP5 (**c’**, *white arrowheads* in **c”’**) in the apical membranes and the cytoplasm; AQP4 (**c”**, *hollow arrowheads* in **c”’**) in the basolateral membranes of OSCs in the apical turn of the mouse (p14) cochlea (*SP* spiral prominence, *CC* Claudius cells). Scale bars: (**a**), 20 μm; (**c–c”’**), 10 μm

The baso-apical extent of AQP4 and AQP5 immunofluorescence signals in the outer sulcus region was analyzed on epifluorescence images of lateral wall whole-mount specimens using the tool for curved length measurements in the software Axiovision (V. 4.8.2.0, Zeiss). Representative confocal images of AQP4 and AQP5 fluorescence signals in the outer sulcus region were obtained in different focal planes: at the luminal cell surface (apical membranes) and in the depth of the spiral ligament where the OSCs’ root processes (basolateral membranes) are located, respectively. Fluorescence images from both focal planes are merged together in Fig. 1a.

### Ratio of apical membrane-to-cytoplasm distribution of AQP5 immunofluorescence in OSCs

**Fig. 2:**
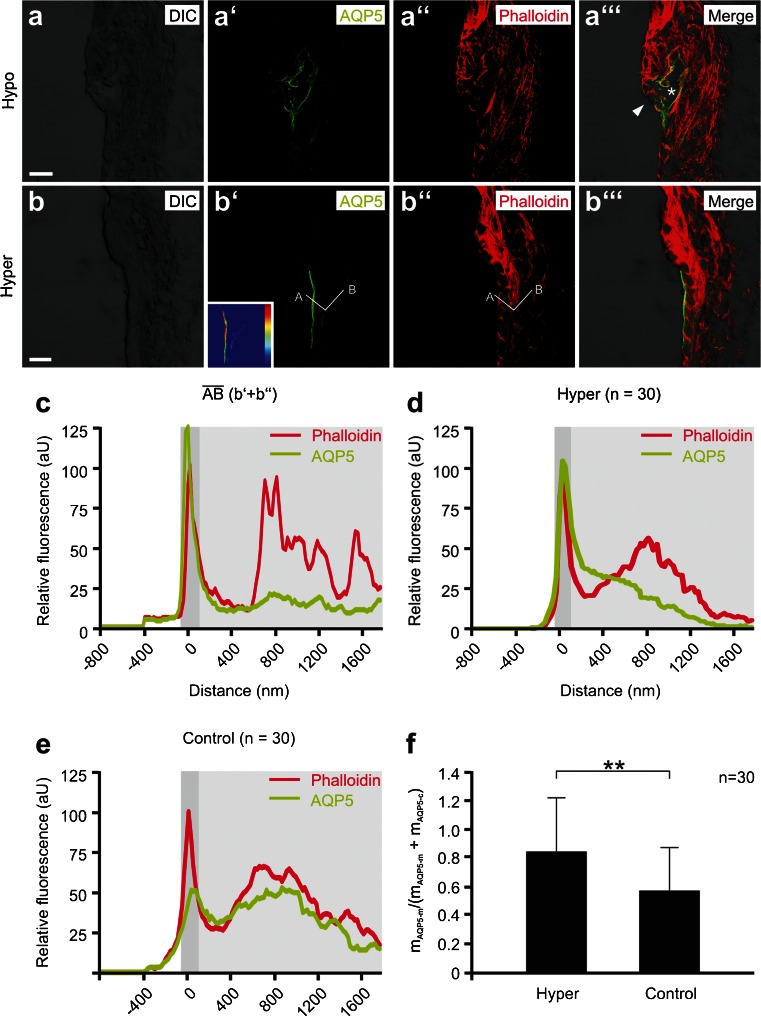
Membrane translocation of AQP5 in outer sulcus cells (OSCs) induced by perilymphatic hyperosmolarity in vitro. **a–a”’** Representative confocal images of AQP5 and phalloidin fluorescence in the spiral ligament from a cochlea that was perilymphatically perfused and incubated with a solution that was hypoosmolar (200 mOsm/L) compared with the endolymph. The overall morphology of the cochlear duct epithelium appeared normal (Supplementary Figure 2); only OSCs in the apical turn showed signs of cellular damage, i.e., disruption of their cytoplasm (*asterisk*, **c”’**) and apical membranes, as indicated by the discontinuity of the phalloidin and AQP5 signals at their luminal cell borders (*white arrowhead*, **c”’**). **b, c** Representative confocal images of AQP5 and phalloidin fluorescence in the spiral ligament from a cochlea that was perilymphatically perfused and incubated with a hyperosmolar solution (400 mOsm/L). The inlay in (**b’**) shows the color-coded AQP5 fluorescence intensity (*dark blue*, low intensity; *dark red*, high intensity) in the OSCs from (**b’**). Along the *white line* $\overline{AB}$ (**b’** and **b”**) that crosses the apical membrane and the cytoplasm of a single OSC, histograms of phalloidin and AQP5 fluorescence intensities (**c**) were generated. The *dark gray-shaded area* in the diagram indicates the apical cell membrane, which was defined by the first peak of phalloidin fluorescence; the *light gray-shaded area* indicates the cytoplasm. **d** Average curves of AQP5 and phalloidin fluorescence intensities derived from 30 measurements from the experimental group “hyperosmolarity” (*n* = 30). **e** Average curves of AQP5 and phalloidin fluorescence intensities derived from 30 measurements from the “isoosmolarity” experimental group (*n* = 30). **f** Quantitative comparison of the m_AQP5m_/(m_AQP5m_ + m_AQP5c_) ratios for the “hyperosmolarity” and “isoosmolarity” experimental groups. *Error bars* indicate the SD one-way ANOVA followed by Tukey’s post hoc test: **p ≤ 0.01; scale bars: 10 μm

For the semi-quantitative analysis of the AQP5 fluorescence signal intensity in the apical cell membrane and the cytoplasm of cochlear OSCs, confocal images were taken from cochlear specimens that were exposed in vitro to different osmolarities or to M3R antagonist/agonists. For acquisition of images that were semi-quantitatively compared with each other, the same microscope configuration and software settings (40-fold (0.8 numerical aperture) oil immersion objective, pinhole diameter, detector gain, and amplifier offset) were applied. Line profiles (white lines $\overline{AB}$ in Figs. 2 and 4) were traced across single OSCs in the LSM 5 PASCAL software (Zeiss) to obtain relative pixel densities for AQP5 and phalloidin fluorescence signals (see example histograms for a single OSC in Fig. 2c). The two *x*-axis positions at the half maximum of the first peak of phalloidin fluorescence (*y*-axis values) were used to define the area of the apical membrane of OSCs (dark gray-shaded areas in the diagrams, Figs. 2 and 4). AQP5 fluorescence signals between the area of the apical membrane and endpoint “B” of the line profile were considered cytoplasmic (light gray-shaded areas in the diagrams, Figs. 2 and 4). Line profiles from 30 OSCs (*n* = 30) were captured per experimental condition. Numerical data from the line profiles were analyzed using Microsoft Excel 2013 (Microsoft, Redmond, WA, USA). The *x*-axis dependent mean values of AQP5 and phalloidin fluorescence were determined for each experimental condition and plotted in Figs. 2d, e and 4d–g. From these data, the mean values of AQP5 fluorescence intensity in the apical cell membrane (m_AQP5-m_, mean value of AQP5 fluorescence intensity in the dark gray-shaded diagram area) and the cytoplasm (m_AQP5-c_, mean value of AQP5 fluorescence intensity in the light gray–shaded diagram area), as well as the standard deviations (SD_AQP5-m_ and SD_AQP5-c_) were determined. The ratios of the mean AQP5 fluorescence intensity in the apical membrane over the total AQP5 fluorescence intensity (apical membrane and cytoplasm) in the cell (m_AQP5-m_/(m_AQP5-m_ + m_AQP5-c_)) and the corresponding SDs were calculated using:

$$ \sqrt{{\left(\frac{{\mathrm{SD}}_{AQP5-m}}{m_{AQP5-m}}\right)}^2+{\left(\frac{{\mathrm{SD}}_{AQP5-m}+{\mathrm{SD}}_{AQP5-c}}{m_{AQP5-m}+{m}_{AQP5-c}}\right)}^2}\times \frac{m_{AQP5-m}}{m_{AQP5-m}+{m}_{AQP5-c}}. $$

### Statistical analysis

Values for the longitudinal extent of AQP4 and AQP5 expression are presented as the means with standard deviations (SD; Fig. 1b), and statistical comparisons using the unpaired Student’s *t* test were performed in Microsoft Excel 2013.

**Fig. 3:**
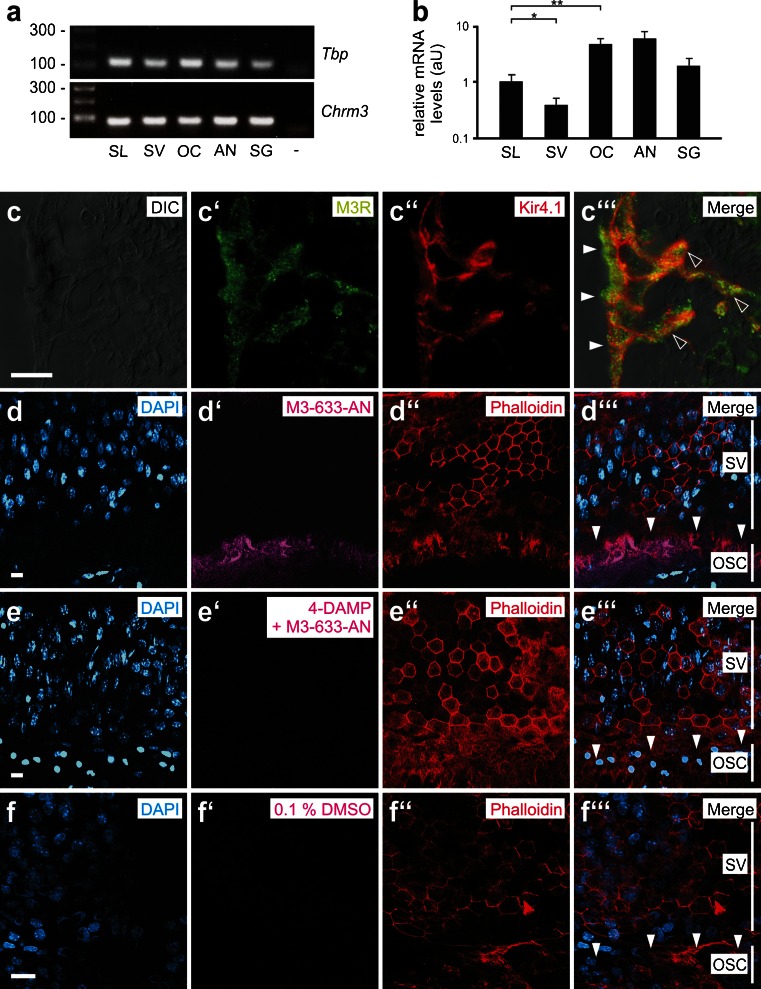
Gene expression, immunolocalization, and in vitro ligand binding of the muscarinic (M3) acetylcholine receptor (M3R) in outer sulcus cells (OSCs) of the mouse (p14) cochlea. **a, b** Quantitative real-time PCR (qRT-PCR) data for the M3R expression in tissue samples from the mouse (p14) cochlea and positive control tissue (*SL* spiral ligament, *SV* stria vascularis, *OC* organ of Corti, *AN* auditory nerve with spiral ganglia, *SG* salivary (parotid) gland, - negative control). *Error bars* indicate the SD, Student’s *t* test: **p ≤ 0.01, *p ≤ 0.05. **c–c”’** Immunolabeling of M3R and the inward rectifier type potassium channel Kir4.1 in OSCs in the apical cochlear turn. M3R fluorescence was detected in the soma region (*white arrowheads*) and root processes (*hollow arrowheads*) of OSCs. **d–f”’** Spiral ligament whole-mount specimens that were directly **d–d”’** incubated with the M3R fluorescent ligand M3-633-AN, incubated with M3-633-AN after **e–e”’** pre-incubation with the M3R antagonist 4-DAMP, or **f–f”’** incubated with 0.1 % DMSO. *Vertical white lines* in **d”’, e”’,** and **f”’** indicate the radial extent of the stria vascularis (SV) and the outer sulcus cells (OSC); *white arrowheads* **d”’, e”’** and **f”’** point to the M3-633-AN fluorescence in the area of the **d”’** OSCs, which is absent in **e”’** and **f”’**. Scale bars: **c–c”’** 10 μm; **d–f”’** 20 μm

The qRT-PCR data were evaluated using ANOVA, and statistical comparisons were performed using qBasePlus software (Biogazelle) (Supplementary Table 1). Finally, the data were calibrated to the biological reference group. Data are presented as the means with SDs (Fig. 3b).

**Fig. 4:**
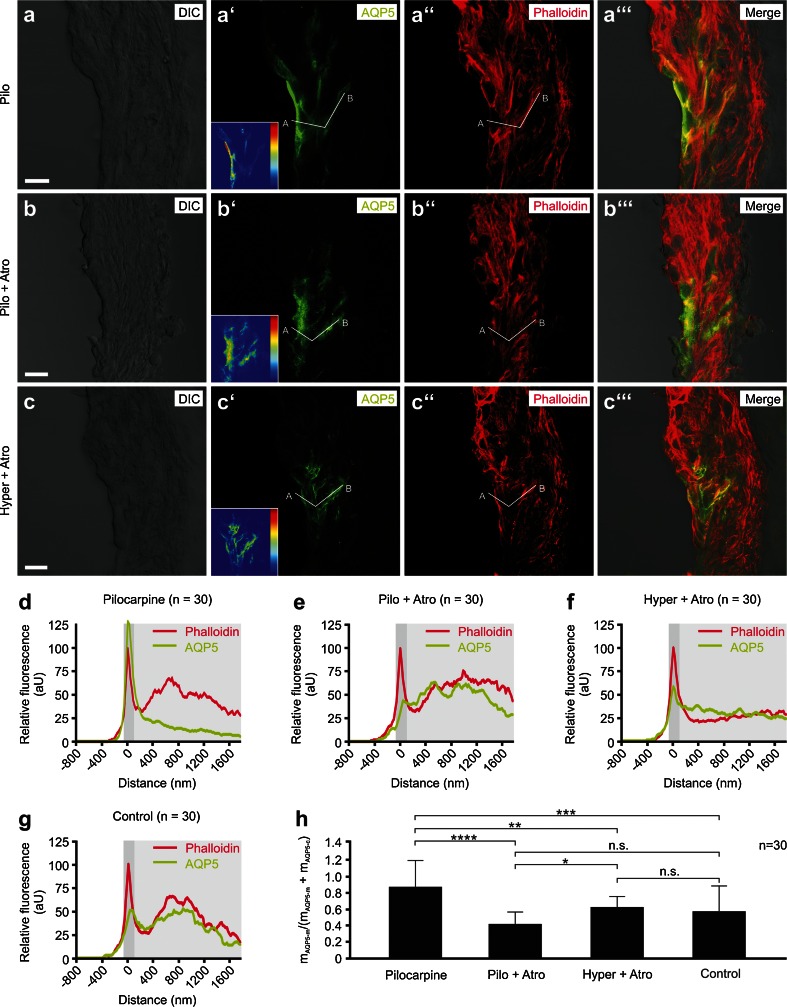
Membrane translocation of AQP5 in outer sulcus cells (OSCs) in vitro is controlled by muscarinic (M3) acetylcholine receptors. **a–c”’** Representative confocal images of AQP5 and phalloidin fluorescence in the spiral ligament of the apical turn of cochlear specimens from the “pilocarpine” (**a–a”’**), “pilocarpine + atropine” (**b–b”’**), and “hyperosmolarity + atropine” (**c–c”’**) experimental groups. Inlays in (**a’**), (**b’**), and (**c’**) show the color-coded AQP5 fluorescence intensity (*dark blue* low intensity; *dark red* high intensity) in OSCs. *White lines* $\overline{AB}$ in (**a’**), (**a”**), (**b’**), (**b”**), (**c’**), and (**c”**) were used to generate histograms of phalloidin and AQP5 fluorescence intensities for single OSCs. **d–g** Average curves of AQP5 and phalloidin fluorescence intensities derived from 30 measurements (*n* = 30) from the pilocarpine, pilocarpine + atropine, hyperosmolarity + atropine, and isoosmolarity experimental groups. The *dark gray-shaded area* in the diagrams indicates the apical cell membrane, which was defined using the first peak of phalloidin fluorescence; the *light gray-shaded area* indicates the cytoplasm. **h** Quantitative comparison of the m_AQP5m_/(m_AQP5m_ + m_AQP5c_) ratios that were calculated based on the data shown in **d**–**g**. Relative fluorescence intensity (rel. FI) in **d–g** is shown in arbitrary units (aU). *Error bars* indicate the SD, one-way ANOVA followed by Tukey’s post hoc test: *n.s.* no statistical significance; *p ≤ 0.05; **p ≤ 0.01; ***p ≤ 0.001; ****p ≤ 0.0001. Scale bars: 10 μm

The ratios m_AQP5-m_/(m_AQP5-m_ + m_AQP5-c_) from different experimental groups were compared for statistical significance using a one-way analysis of variance (ANOVA) and the Tukey’s honestly significant difference (HSD) post hoc test to detect statistically significant differences between groups. Ratios m_AQP5-m_/(m_AQP5-m_ + m_AQP5-c_) are presented as the means with SD (Figs. 2f and 4h).

For all statistical data presented, *p* values ≤0.05 were considered statistically significant.

## Results

### Postnatal development of AQP4 and AQP5 expression and subcellular localization in OSCs of the murine cochlea

The baso-apical extent of AQP4 and AQP5 expression in the outer sulcus region was measured on whole-mount specimens of the lateral wall from p0 to p128 NMRI mouse cochleae (Fig. 1a, b). The length measurements of AQP4 immunofluorescence labeling revealed a sudden onset of AQP4 expression in the outer sulcus region of all cochlear turns between p8 (0 mm) and p10 (6.8 ± 0.06 mm); thereafter, the longitudinal extent of AQP4 expression in the outer sulcus region remained constant into the late adult stages (p128). Notably, in the inner sulcus region and the greater epithelial ridge (GER), respectively, AQP4 was labeled as early as p2 (data not shown). In contrast to AQP4, AQP5 expression was present in the outer sulcus region along the entire length of the lateral wall at birth (p0, 5.15 ± 0.15 mm). This expression pattern remained constant until p4 (6.51 ± 0.07 mm) but rapidly declined in the basal and middle cochlear turns between p8 (5.97 ± 0.07 mm) and p12 (2.64 ± 0.13 mm). A further, but only gradual, decrease in the longitudinal AQP5 expression was identified from p12 to p32 (1.46 ± 0.06 mm), which resulted in a total decline of the longitudinal extent of AQP5 expression of approximately 77.57 % between p4 and p32. Finally, at p32, an overlapping AQP4 and AQP5 expression in the apical cochlear turn was fully established over a distance of 1.46 ± 0.06 mm. Notably, the developmental decline in AQP5 expression along the baso-apical axis of the cochlear duct accompanied the baso-apical morphological maturation of the outer sulcus region in NMRI mice (Supplementary Figure 1).

Immunofluorescence labeling of AQP4 and AQP5 on midmodiolar cryosections of the p14 NMRI mouse cochlea revealed the polarized localization of AQP4 in the basolateral membranes and AQP5 in the apical membranes of the OSCs. Additionally, strong cytoplasmic AQP5 immunofluorescence was detected in the OSCs (Fig. 1c–c”’). Consistent with previous reports, immunolabeling of AQP4 and AQP5 in positive control tissues (murine kidney and salivary gland for AQP4 and AQP5, respectively) showed the polarized localization of these AQP subtypes in the basolateral membranes of the inner medullary collecting duct epithelium (AQP4, Supplementary Figures 7e–7e”’) and in the apical membranes of salivary gland acinar cells (AQP5, Supplementary Figures 6b–6b”’), respectively.

### Perilymphatic hyperosmolarity induces the membrane translocation of AQP5 in OSCs

In cochleae from the experimental group, “hypo-osmolarity” bulging of Reissner’s membrane (two out of three specimens, Supplementary Figures 2a and 2c) was observed, which suggests a net inflow of water into the endolymphatic fluid space driven by the high osmotic gradient directed into the scala media in our in vitro experiments. The overall morphology of the cochlear duct appeared intact in all three specimens from this group, as evaluated using phalloidin-labeled cryosections (Supplementary Figures 2a, 2c and 2e); however, all three cochlear specimens in this experimental group showed morphological damage of the OSCs in the apical turn. In these OSCs, the disruption of the phalloidin fluorescence in the region of their apical membranes (Fig. 2a–a”’ and Supplementary Figures 2b–2b”’, 2d–2d”’ and 2f–2f”’) and the loss of cellular integrity were noted, which suggests cellular damage induced by hypo-osmotic cell swelling. Therefore, for this particular experimental paradigm, the effect of perilymphatic hypo-osmolarity on the subcellular localization of AQP4 and AQP5 in OSCs could not be analyzed. The ratio m_AQP5-m_/(m_AQP5-m_ + m_AQP5-c_) and the corresponding SD we determined for the paradigm “hyperosmolarity” (=0.85 ± 0.36) was significantly higher (*p* ≤ 0.01) compared with the paradigm “isoosmolarity” (=0.57 ± 0.31), which indicates an increase in AQP5 fluorescence in the apical membranes of OSCs induced by perilymphatic hyperosmolarity in vitro (Fig. 2f). Notably, OSCs exhibited great differences of AQP5 fluorescence intensity in their cytoplasm (weak AQP5 fluorescence in particular in their root processes) compared to their apical membrane (brighter AQP5 fluorescence). In some OSCs, the range of AQP5 fluorescence intensity between the cytoplasm and the apical membrane area exceeded the dynamic range of the spectral detector unit of the microscope, leading to spots of saturated pixels in the digital images. Most of these brightness-saturated spots were excluded from the semi-quantitative analysis of AQP5 fluorescence intensity. However, since these saturated spots were mostly observed in the OSC’s apical membrane area in experimental conditions in which the AQP5 membrane translocation was stimulated (experimental groups “pilocarpine” and “hyperosmolarity”), our analysis potentially slightly underestimated m_AQP5-m_/(m_AQP5-m_ + m_AQP5-c_) for these experimental paradigms. No changes of AQP4 labeling in the basolateral membranes of the OSCs were observed between the “hyperosmolarity” and “isoosmolarity” groups (data not shown).

### Muscarinic (M3) acetylcholine receptors are expressed in OSCs in the murine cochlea and bind M3R-specific ligands in vitro

The gene expression of M3R was detected in different parts of the murine (p14) cochlea, including the spiral ligament (SL), the stria vascularis (SV), the organ of Corti (OC), and the auditory nerve with the spiral ganglia (AN) (Fig. 3a). Quantitatively, the highest levels of M3R mRNA were identified in the OC and the AN (Fig. 3b). Standard curves for the probes *Chrm3* (M3R mRNA-specific probe), *Tbp*, *Ubc*, and *Actb* are shown in Supplementary Figure 3. At the protein level, M3R immunolabeling was detected in the OSCs that were attached to the SL (Fig. 3c–c”’), in the SV (Supplementary Figures 4a–4a”’), in the spiral ganglion (SGg) cells (Supplementary Figures 4b–4b”’), and in the central part of the AN (Supplementary Figures 4c–4c”’). Omitting the M3R primary antibody or pre-incubation of the primary antibody with the corresponding control peptide resulted in a complete absence of M3R immunolabeling in all cochlear turns and positive control (parotid gland) tissues (data not shown). To test whether M3R ligands bind in vitro to M3Rs in OSCs, we incubated whole-mount specimens of the murine (p14) spiral ligament with the specific fluorescent M3R ligand M3–633–AN (100 nM) alone or after pre-incubation with the unlabeled M3R competitor 4-DAMP (10 μM). Microscopic analysis of the phalloidin and DAPI-labeled specimens revealed a stripe of M3–633–AN fluorescence beneath the stria vascularis in the outer sulcus region (Fig. 3d–d”’). In specimens that were pre-incubated with 4-DAMP (Fig. 3e–e”’) or that were incubated in 0.1 % DMSO only (Fig. 3f–f”’), no M3–633–AN fluorescence was detected. The microscopic focal plane for the detection of M3–633–AN fluorescence in OSCs was chosen in the depth of the spiral ligament whole-mount specimens, whereas the focal plane to capture phalloidin and DAPI fluorescence signals was set at the surface of the whole-mount specimens. The binding of the fluorescent M3R ligand M3–633–AN in the outer sulcus region and its competitive inhibition by 4-DAMP in vitro confirms the presence of M3Rs in OSCs. Therefore, we postulated that the incubation of spiral ligament whole-mount specimens with M3R antagonist/agonists enables the stimulation or inhibition of M3Rs in OSCs in vitro.

### Pilocarpine induces the membrane translocation of AQP5 in OSCs

Semi-quantitative analysis of AQP5 immunofluorescence in the apical membrane and the cytoplasm of the OSCs from the ‘pilocarpine’ (Fig. 4a–a”’), ‘pilocarpine + atropine’ (Fig. 4b–b”’), and ‘hyperosmolarity + atropine’ (Fig. 4c–c”’) experimental groups was performed as previously described for the cochleae that were exposed to iso- and hyperosmolar solutions. The average curves of AQP5 fluorescence intensity in the apical membrane and the cytoplasm derived from 30 measurements per experimental condition (*n* = 30) are shown in Fig. 4d–g. A comparison of the m_AQP5-m_/(m_AQP5-m_ + m_AQP5-c_) values from the “pilocarpine” (= 0.86 ± 0.33), “pilocarpine + atropine” (= 0.41 ± 0.15), “hyperosmolarity + atropine” (= 0.62 ± 0.11), and “isoosmolarity” (= 0.57 ± 0.31) groups is shown in Fig. 4h. In the OSCs from the pilocarpine group, a strong co-localization of immunofluorescence signals for AQP5 and the lipid raft-associated integral membrane protein Flot-2 was found in the apical membrane region, whereas nearly no AQP5 and Flot-2 immunofluorescence signals were detected in the cytoplasm of the OSCs (Supplementary Figures 6a–6a”’).

Representative images from parotid gland tissue slices from the pilocarpine (Supplementary Figures 6b–6b”’) and pilocarpine + atropine (Supplementary Figures 6c–6c”’) groups showed strong AQP5 immunofluorescence at the apical pole of acinar cells in the pilocarpine group, whereas in the pilocarpine + atropine group, the AQP5 fluorescence signal was less intense at the apical pole and distributed throughout the cytoplasm. This positive control confirms the M3R-regulated translocation of AQP5 in acinar cells in vitro, as previously reported [41, 56].

### Aquaporins 4 and 5 and muscarinic (M3) acetylcholine receptors are expressed in OSCs in the human cochlea

**Fig. 5:**
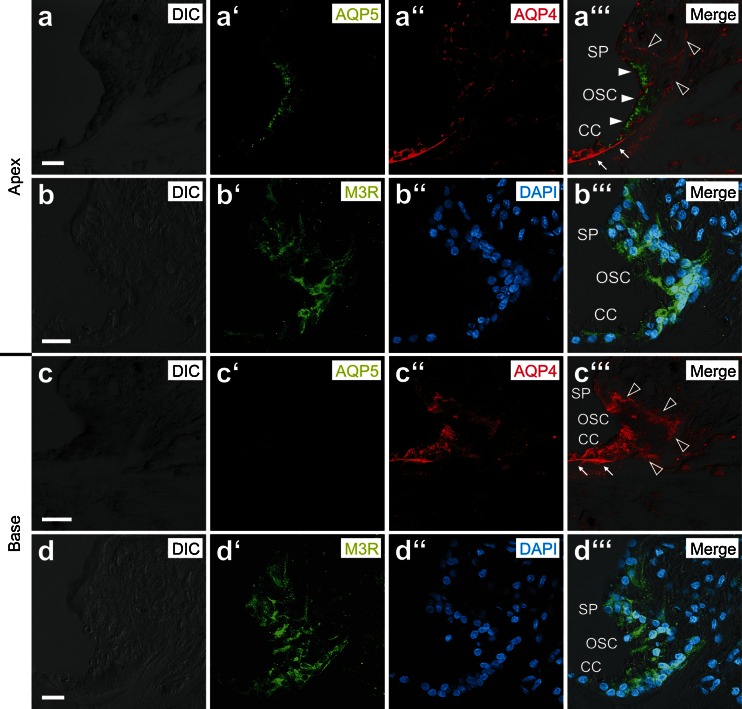
Immunolocalization of AQP4, AQP5, and M3R in outer sulcus cells (OSCs) of the adult human cochlea. **a–b”’** In the apical turn, AQP4 labeling was localized in the basolateral membranes (*hollow arrowheads*, **a”’**), and AQP5 labeling was detected in the apical membranes and the subapical cytoplasm (*white arrowheads*, **a”’**) of OSCs. M3R labeling was also detected in OSCs in the apical cochlear turn (**b’–b”’**). **c–d”’** In the basal turn, OSCs exhibited AQP4 labeling in their basolateral membranes (*hollow arrowheads*, **c”’**) but were devoid of AQP5 labeling. OSCs in the basal cochlear turn were also immunoreactive for M3R (**d–d”’**) (*White arrows* in (**a”’** and **c”’**) indicate AQP4 labeling in the basal membranes of Claudius cells (CCs) in the apical (**A”’**) and basal turn (**C”’**)). (*SP* spiral prominence). Scale bars: 20 μm

Immunolabeling for AQP4, AQP5, and M3R in the human cochlea revealed a cellular and subcellular localization pattern in the OSCs that was identical to that in the OSCs in the mouse cochlea. The OSCs in the apical turn of the human cochlea showed a complementary membranous localization of AQP4 (basolateral) and AQP5 (apical) (Fig. 5a–a”’), as well as immunolabeling for M3R (Fig. 5b–b”’). The OSCs in the basal cochlear turn were labeled for AQP4 (Fig. 5c–c”’) and M3R (Fig. 5d–d”’) but were devoid of AQP5 labeling. The results from the positive control experiments for AQP5 and M3R labeling on human salivary (parotid) gland tissue slices are shown in Supplementary Figure 7.

## Discussion

### The complementary co-localization of AQP4 and AQP5 in the outer sulcus cells in the cochlear apex emerges at the onset of hearing function

**Fig. 6:**
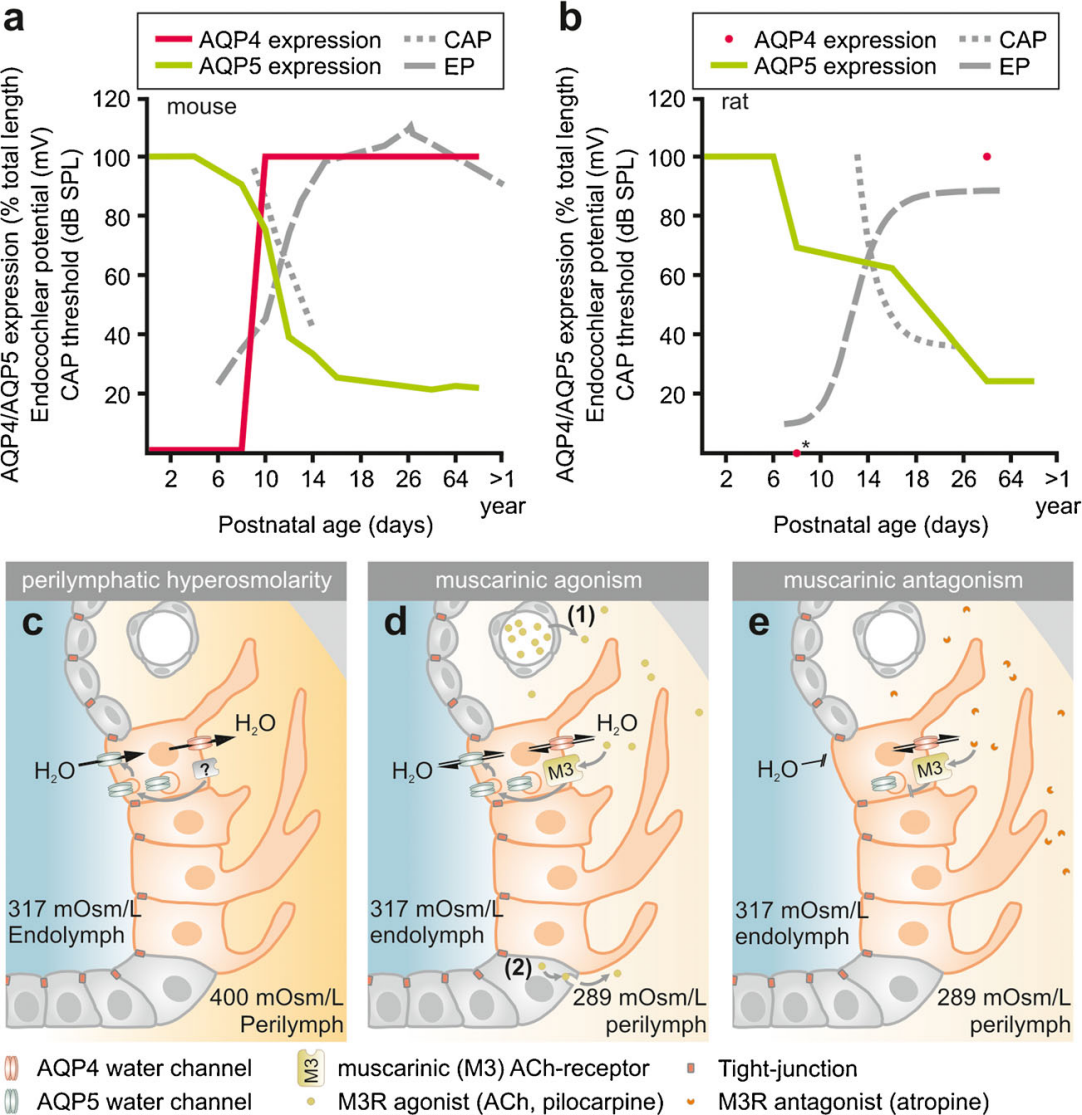
**a, b** In the postnatal development of the mouse (**a**) and the rat cochlea (**b**), the baso-apical decline of AQP5 expression in OSCs (*green curves*) and the onset of AQP4 expression (*red curve* in **a**, *red dots* in **b**) temporally coincide with the formation of the endocochlear potential (*EP*, *black dashed lines*) and the lowering of the compound action potential (*CAP*) thresholds (*black dotted lines*) around p10. CAP thresholds are provided for the 5-kHz frequency. EP and CAP data in **a** and **b** were reprinted from [7] and [64] with the kind permission of the publishers of *Informa Healthcare* and *Elsevier*); *asterisk* data point is based on our unpublished data. **c–e** Schematic illustration of AQP5 apical membrane translocation in OSCs (*orange cells*) in response to perilymphatic hyperosmolarity (**c**), muscarinic (M3R) agonism (**d**), and its internalization into the cytoplasm induced by muscarinic (M3R) antagonism (**e**). The cellular signaling cascade by which extracellular osmolarity changes induce AQP5 membrane translocation remain unknown (? in **c**). The source of physiological M3R agonists that stimulate AQP5 membrane translocation in OSCs could be the blood plasma [considering the dense vascularization of the spiral ligament ((*1*) in **d**)) or cells of the cochlear duct ((*2*) in (**d**)), which are a source for non-neuronal acetylcholine (ACh) that would act on M3Rs in OSCs in a paracrine manner. Direct M3R antagonism (**e**) blocks the membrane translocation of AQP5 water channels in OSCs. Therefore, the reduced water permeability of the apical membrane domain restricts transcellular water movements across OSCs. Note that AQP4 constitutively facilitates water movements across the basolateral membrane domain of OSCs (**c–e**). *Black arrows* indicate the directions of putative transcellular water flow

During the postnatal development of the murine (NMRI mice) cochlea, AQP4 and AQP5 show distinct spatiotemporal expression patterns in OSCs along the baso-apical axis of the cochlear duct. A sudden onset of AQP4 expression in OSCs at all cochlear turns was observed between p8 and p10; in contrast, in the epithelial cells of the GER/inner sulcus region, the onset of AQP4 expression was demonstrated as early as p2 (data not shown), which is consistent with the results of a previous study in which AQP4 mRNA expression was detected in the murine (CD-1 mice) cochlea at p1 [36]. Our immunohistochemical results therefore indicate a developmental gradient for the onset of AQP4 expression along the radial axis of the cochlear duct. At early postnatal ages (p0, p2), the OSCs of all cochlear turns expressed AQP5 that was rapidly lost between p4 and p12 along the baso-apical axis and finally became restricted to the OSCs in the most apical 1.46 ± 0.06 mm of the cochlear duct at p32. This longitudinal decline in AQP5 expression was accompanied by the relatively late morphological maturation of the outer sulcus, i.e., the overgrowth of OSCs by CCs in the basal and middle cochlear turns. Similar spatiotemporal changes in the outer sulcus with a decrease in the longitudinal extent of AQP5 expression of 65.11 % and overgrowth of OSCs by CCs were previously described in the rat (Wistar rats) cochlea [8, 33]. These spatiotemporal changes in AQP4 and AQP5 expression in OSCs in the mouse (Fig. 6a) and rat (Fig. 6b, data from [33]) cochleae converge in the formation of a perilymphatic–endolymphatic “water shunt” in the cochlear apex [19, 21, 33] and parallel the functional maturation of the hearing organ, i.e., an increase in the endocochlear potential (EP) and a reduction in compound action potential (CAP) thresholds [7, 64].

The longitudinal extent of the overlapping membrane expression of AQP4 (basolateral) and AQP5 (apical) at an adult-like age (p128) represented 22.32 % of the total length of the murine (NMRI) cochlear duct, which is consistent with the previously measured extent of AQP5 expression in the rat (Wistar rats; p128) cochlear duct (29.1 % of the total length [33]) but considerably longer compared with the extent of overlapping AQP4/AQP5 expression in the adult guinea pig (strain BFA bunt) cochlear duct (3.97 % of the total length [21]). These marked differences in the surface area of the AQP4/AQP5–water shunt in the cochlear apex between rodent species potentially reflect different physiological requirements for water homeostasis in the cochlea. Such species-specific differences have already been proposed based on results from in vivo experiments in which the endolymphatic duct and sac (portions of the inner ear epithelium that presumably act in inner ear fluid regulation) had been surgically destroyed [52, 61]; guinea pigs reliably developed an overaccumulation of endolymphatic fluid (i.e., endolymphatic hydrops) within days to weeks after surgery, whereas rats were much less susceptible to this ablative treatment and developed an endolymphatic hydrops in only about half of the cases.

### Disturbances in perilymphatic osmolarity induce the apical membrane translocation of AQP5 water channels and challenge cell survival in outer sulcus cells in the cochlear apex

In the present study, an in vitro induced increase in the perilymphatic osmolarity to 400 mOsm/L for 10 min resulted in a significant increase in AQP5 immunofluorescence in the apical membrane of OSCs and a reduced AQP5 fluorescence in the cytoplasm, which suggests a tonicity-induced membrane translocation of AQP5 water channels. Furthermore, an in vitro decrease in the perilymphatic osmolarity to 200 mOsm/L for 10 min resulted in a selective cellular damage of AQP4/AQP5-expressing OSCs in the cochlear apex with morphologically determined loss of their apical membrane barrier.

Changes in extracellular osmolarity are an established trigger for the regulation of AQP membrane abundance. In different in vitro and in vivo models, hypo-osmolarity induced the membrane translocation of AQP1, AQP3, and AQP8, whereas hyperosmolarity induced the membrane translocation of AQP1–5 and AQP9 [reviewed in 16]. The regulation of AQP membrane abundance by osmotic gradients enables cells to rapidly and selectively adjust the water concentration in their cytosols and the surrounding extracellular compartments to the prevailing physiological requirements.

In the cochlea, differences in the ionic concentrations [77] and osmolarities are present between the endolymphatic (322.7 mOsm/L (mean of all cochlear turns)) and perilymphatic fluid compartments (298 mOsm/L) [79]. These ionic and osmotic gradients are maintained by molecular ion transport mechanisms [reviewed in 55] and the tight membranous boundaries that seal the endolymphatic fluid space, designated the “perilymph–endolymph barrier” [28, 44], both of which are established by cells of the cochlear duct. The distinct ionic and osmotic concentrations in the cochlear fluids are continuously challenged by transepithelial ion movements across the cochlear duct epithelium, as well as serum osmolarity changes that are rapidly transferred to the perilymphatic fluid via the relatively permeable blood–perilymph barrier [47, 48, 80]. In humans, serum osmolarities between 241 and 352 mOsm/L have been reported [71]. Changes in perilymph osmolarities within the same range were demonstrated to affect cochlear function in guinea pigs in vivo (260 and 340 mOsm/L, respectively) [15] and cause transiently increased thresholds of stimulus-related cochlear potentials, specifically at low frequencies (262 mOsm/L [46]). Salt and DeMott [76] demonstrated in vivo that osmolarity changes in the perilymphatic fluid induce LEMs, which in turn account for the correction of osmolarity-induced endolymphatic volume disturbances. In a recent study in which we investigated the molecular basis of LEMs in the cochlea, we determined a remarkably high osmotic water permeability (osmotic water permeability coefficient (P_f_) of 156.90 × 10^−3^ cm/s) for OSCs in the cochlear apex during perilymphatic hyperosmolarity; we proposed a model explaining LEMs based on osmotically driven water flow through AQP4 and AQP5 in these OSCs [21]. The results of the present study support this model by indicating that perilymphatic hyperosmolarity increases the membrane abundance of AQP5 in OSCs and thereby potentially facilitates water movements from the endolymphatic to the perilymphatic fluid compartment along the prevailing osmotic gradient (Fig. 6c).

In contrast, perilymphatic hypoosmolarity (200 mOsm/L, 10 min) in vitro resulted in histologically discernable endolymphatic hydrops (in two out of three specimens), which suggests a net water flow into the endolymphatic fluid space across the perilymph–endolymph barrier. According to our previous model [21], perilymphatic hypo-osmolarity forces water to flow across AQP4/AQP5-expressing OSCs in the cochlear apex into the endolymphatic fluid space, which results in apico-basally-directed LEMs. In our experiments, a reduction in perilymphatic osmolarity to 200 mOsm/L caused histologically determined cell damage that was restricted to AQP4/AQP5-expressing OSCs in the cochlear apex (in three out of three specimens). This finding may be explained by the constitutively high osmotic water permeability of the large, AQP4-expressing basolateral membranes of OSCs, which in our experiments enabled excess cellular water entry and consecutive osmotic cytolysis. Morphologically, the hypo-osmolarity-induced disruption of OSCs was consistent with a local breakdown of the perilymph-endolymph barrier in the cochlear apex. This loss of barrier function in OSCs might account for the incomplete recovery of the EP and the endolymph volume after perilymphatic perfusion with hypo-osmolar (relative to the endolymph osmolarity) solution, as observed by Salt and DeMott [76].

In conclusion, we show in the present study that AQP5 in AQP4/AQP5-expressing OSCs in the cochlear apex is dynamically regulated by changes in perilymphatic osmolarity. The hyperosmolarity-induced membrane translocation of AQP5 presumably raises their transcellular water permeability and enables OSCs to mediate osmotically driven water movements that induce LEMs and are crucial for cochlear fluid homeostasis.

### Autonomic (muscarinic) activation induces the apical membrane translocation of AQP5 water channels in outer sulcus cells in the cochlear apex

In the present study, M3R mRNA expression in the lateral wall, M3R immunolocalization in OSCs, and in vitro mapping of M3R with a receptor-specific fluorescent ligand confirmed the presence of M3Rs in AQP4/AQP5-expressing OSCs. The in vitro application of the M3R agonist pilocarpine (10 μM) significantly increased AQP5 immunofluorescence in the apical membranes of OSCs, whereas this effect was blocked by the simultaneous application of the M3R antagonist atropine (100 μM). Additionally, after pilocarpine treatment, a strong membrane colocalization of immunofluorescence signals for AQP5 and flotillin-2—a marker for specialized membrane domains that are involved in vesicular trafficking—in OSCs suggests the pilocarpine-induced fusion of AQP5-containing intracellular membrane vesicles with the apical cell membrane. These results indicate a M3R-mediated membrane translocation mechanism in AQP4/AQP5-expressing OSCs in the cochlear apex.

An M3R-mediated apical membrane translocation of the AQP5 water channel has been shown or discussed for several AQP5-expressing secretory tissues, including salivary [24, 25, 41, 42, 56], lacrimal [39], and sweat glands [38, 68, 78], as well as the lung alveolar epithelium (proven cAMP-dependent translocation cascade [88, 89]). In these tissues, autonomic (muscarinic) stimuli increase the AQP5-mediated transepithelial fluid flow via M3R activation and thereby increase the secretion rate for saliva, tear fluid, sweat, and mucus.

In the cochlea, endolymphatic K^+^ secretion by the stria vascularis, which is required for the maintenance of the EP and a constant volume of endolymph, is stimulated by β1 adrenergic agonists and inhibited by M3 and/or M4 muscarinic agonists [86, 87]. This finding suggests an autonomic regulation of endolymph production in the cochlea. Based on the present study, we propose to extend the muscarinic regulation of AQP5 mediated water flow to the OSCs (Fig. 6d, e). Interestingly, OSCs were previously established as a parasensory pathway for the resorption of cations from endolymph (K^+^, Na^+^) based on their apical membrane expression of potassium channels (BK channels) and chloride/iodide transporters (pendrin) [13, 14, 43, 57, 62]. This finding suggests that cholinergic (muscarinic) stimuli in the cochlea can downregulate the endolymph production by the stria vascularis and simultaneously upregulate the water permeability of the OSCs. Moreover, muscarinic stimuli may also regulate the activity of ion channels and transporters in OSCs in order to control parasensory ion reabsorption from the endolymphatic fluid. Taken together, the concerted muscarinic effects on stria vascularis cells and OSCs are presumably of significance for the maintenance of a constant endolymph volume.

Moreover, in the present study, we demonstrated that AQP5 membrane translocation in OSCs induced by the perilymphatic application of a hyperosmolar solution was partially blocked by atropine. It remains unclear how M3R antagonism interferes with the cellular signaling cascade that mediates perilymphatic hyperosmolarity-induced AQP5 membrane translocation in OSCs; however, other examples of the inhibition of hyperosmolarity-induced cellular effects by blocking G-protein coupled receptors (GPCRs) or targets in their respective intracellular signaling cascade (e.g., the blockade of neurokinin 3 receptors (NK3R) in the paraventricular nucleus of the hypothalamus inhibits the hyperosmolarity-induced release of vasopressin [29]), and the blockade of phosphokinase A (PKA) inhibits the vasopressin-2 receptor (V2R)-mediated apical membrane translocation of AQP2 in cultured renal-collecting duct principal cells [30].

In conclusion, in the present study, we provide evidence for the dynamic regulation of AQP5 localization in AQP4/AQP5-expressing OSCs by muscarinic agonists/antagonists via M3R. The M3R-controlled AQP5 membrane translocation in OSCs in the cochlear apex presumably regulates their transcellular water permeability and constitutes a regulatory mechanism for the perilymphatic–endolymphatic water homeostasis by autonomic stimuli.

### Potential implications for the regulated aquaporin-based water shunt in endolymphatic hydrops and Ménière’s disease

In the present study, we demonstrated that the molecular determinants of the regulated AQP-based water shunt (AQP4, AQP5, and M3R) are also expressed in OSCs in the apex of the human cochlea. Therefore, failure in the human cochlear AQP4/AQP5–water shunt could contribute to impaired endolymph volume homeostasis, e.g., leading to the endolymphatic hydrops in Ménière’s disease.

In Ménière’s disease, the generation of endolymphatic hydrops has been attributed to osmotic imbalances between the cochlear fluid compartments [54] and to the stagnation of LEMs that putatively counteract these imbalances [27, 76]. We have shown that the perilymphatic osmolarity changes that induce LEMs in vivo [76] regulate the membrane abundance of AQP5 water channels in OSCs in the cochlear apex. The dysregulation of this AQP-based water shunt may potentially impair LEMs and thereby contribute to the generation of the endolymphatic hydrops in Ménière’s disease. This dysregulation could manifest in two ways: (i) the perilymphatic–endolymphatic osmotic gradient, which constitutes the driving force for transcellular water movements across the AQP4/AQP5–water shunt, is disturbed and/or (ii) the AQP-based transcellular water permeability of the AQP4/AQP5–water shunt is altered. In line with (i), altered serum osmolarities have been reported in Ménière’s patients in acute attacks and during remission phases [5, 11, 12, 50, 53, 81]. Moreover, in the clinical diagnosis of Ménière’s disease, the sugar alcohol glycerol is administered orally or intravenously to increase the serum and perilymph osmolarity and thereby osmotically drain the hydropic endolymphatic fluid space and temporarily improve audiometric hearing thresholds (in the early disease state when endolymphatic hydrops remains reversible) [54]. This finding implies that endolymphatic hydrops in Ménière’s disease is a consequence of osmotic imbalances that can be temporally compensated by osmotically forced (induction of perilymphatic hyperosmolarity) water outflow from the endolymph across the boundaries of the cochlear duct epithelium, as demonstrated in vivo [17, 18, 49, 65, 82]. In line with (i) and (ii), the serum (and perilymph) osmolarity [9] and the AQP5 membrane abundance in OSCs (present study) are both controlled by the autonomic system. Autonomic dysregulation has been proposed as a contributing factor to Ménière’s disease based on elevated serum levels of stress hormones [6, 35, 70, 85] and hyperreactivity of peripheral cholinergic receptors [63], as well as abnormal pupillary reflexes in response to muscarinic stimulation (methacholine test) [37, 83, 84] and sympathetic hypofunction [90] homolateral to the diseased inner ear in Ménière’s patients. Therefore, autonomic dysregulation in Ménière’s disease could not only account for pathological serum (and perilymph) osmolarity changes but also alter the cholinergic (M3R)-controlled AQP5 membrane abundance in OSCs that presumably determines its high transcellular water permeability ([21], present study). Finally, in line with (ii), several naturally occurring AQP5 single nucleotide polymorphisms (SNPs)/mutations have been reported to affect the membrane targeting of AQP5 water channel proteins [66] and the transcription rate of the AQP5 gene [1] or to render AQP5 water channels leaky and/or more sensitive to hypotonicity [10]. These AQP5 gene SNPs/mutations are of potential clinical significance because they are associated with decreased saliva secretion (in rats [66]), altered responses of the renin–angiotensin–aldosterone system (RAAS) to salt loading [1], and the occurrence of palmoplantar keratoderma Bothnia type in humans [10]. Interestingly, another AQP5 SNP (variant G allele of rs3736309) was recently associated with a reduced risk for Ménière’s disease [69]; however, the impact of this SNP on the single channel–water permeability and membrane trafficking of the AQP5 channel proteins is unknown. It is also not known whether the abovementioned pathologies/mutations affect the membrane trafficking and/or water permeability of AQP5 channels in cochlear OSCs. AQP5-deficient mice do not exhibit an auditory phenotype (based on auditory brainstem response (ABR) measurements) [59], which suggests that at least under normal conditions other mechanisms may compensate for AQP5 function in the cochlea and thereby maintain endolymphatic fluid homeostasis. In contrast, AQP4-deficient mice show a severe sensorineural hearing loss [59]; however, this cannot be attributed to a dysfunction of the AQP4/AQP5-water shunt in cochlear OSCs, since AQP4 is also abundantly expressed in many other supporting cell types of the cochlear duct [20, 32]. To elucidate the physiological role and pathologic implications of the cochlear AQP–water shunt in endolymphatic volume homeostasis, further studies using in vivo approaches are required.

In conclusion, disturbances in the osmotic driving forces for water movements across the AQP4/AQP5–water shunt and the dysregulation of AQP5-based water permeability may both originate from the autonomic dysregulation that is associated with Ménière’s disease and contributes to the generation of endolymphatic hydrops.

In summary, we provide morphological evidence for a molecular water shunt based on the membrane localization of AQP4 (basolateral) and AQP5 (apical) in OSCs in the murine cochlear apex that develops at the onset of hearing function. The abundance of AQP5 water channels was increased in vitro by perilymphatic hyperosmolarity and muscarinic (M3) receptor stimulation in OSCs, which indicates the regulation of transcellular water permeability in the OSCs by osmolarity changes in the cochlear fluids and autonomic stimuli. We also revealed the molecular determinants of this regulated water shunt to be expressed in OSCs in the apex of the human cochlea. We postulated a potential function of this regulated water shunt in endolymph volume homeostasis and speculated about an affection of its regulated water permeability in Ménière’s disease.

## Electronic supplementary material

Supplementary Figure 1 Morphology of the outer sulcus region and AQP5 expression in outer sulcus cells (OSCs) in the apex and the base of the early postnatal (p4) and mature (p38) murine cochlea. (**a–c**) At p4, AQP5 (green) is expressed in OSCs in the base (a), middle (b), and apex (c) of the cochlea (DAPI, blue) (also see Fig. 1a and b). (**d–f**) Epon sections of the p4 cochlea demonstrate that OSCs in all three turns are between the Claudius cells (CC) and spiral prominence (SP) epithelial cells and that OSCs in all turns contact the endolymphatic fluid space with their apical membranes. (**g–i**) At p38, AQP5 expression (green) disappears in OSCs in the basal (g) and middle turn (h) and is restricted to OSCs in the cochlear apex (i; DAPI, blue) (also see Fig. 1a and b). (**k–m**) Epon sections of the p38 cochlea reveal that OSCs in the basal and middle turn were overgrown by CCs and therefore lost physical contact with the endolymphatic fluid space. In the outer sulcus region in the cochlear apex, the early postnatal morphological configuration is preserved, with AQP5-expressing OSCs between the CCs and SP epithelial cells. Thus, this subpopulation of OSCs retains its direct contact to the endolymphatic fluid space. (SV, stria vascularis). Scale bars: 20 μm. (JPEG 2888 kb)

Supplementary Figure 2 Cellular damage in OSCs in the apical cochlear turn induced by perilymphatic hypoosmolarity. (**a, c** and **e**) Overview of the cochlear duct in the apical turn from the three specimens in the ‘hypoosmolar’ group. Bulging of Reissner’s membrane into scala vestibuli was observed in specimens 1 and 2 (hollow arrowheads, (a) and (c)), which suggests osmotic-driven water flow into the endolymph fluid space. (**b–f”’**) Higher magnification of the outer sulcus area from specimens 1 (b**–**b”’), 2 (d–d”’), and 3 (f**–**f”’). Apical membranes of OSCs are disrupted as indicated by the loss of phalloidin fluorescence at the luminal membrane borders (white arrowheads in (b”), (d”) and (f”)). The neighboring spiral prominence (SP) epithelial cells and Claudius cells (CC) show intact cellular outlines based on their membranous phalloidin fluorescence. Scale bars: (a, c, e), 100 μm; (b**–**b”’, d**–**d”’, f**–**f”’), 10 μm. (JPEG 4370 kb)

Supplementary Figure 3 Primer efficiencies and errors were determined using standard curve experiments on murine (p4) organ of Corti cDNA (for reference genes) or murine (p14) spinal cord cDNA (for *Chrm3*) in a dose-dependent fashion (8 pg–25 ng of cDNA per reaction). The C_T_ value corresponds to the cycle of amplification in which the fluorescence of a sample surpassed the background fluorescence, and the C_T_ values were determined using the LightCycler® 480 Software release 1.5.0 SP4 (Roche Diagnostics). Mean C_T_ value ± SD (triplicates). (JPEG 211 kb)

Supplementary Figure 4 Immunolocalization of the muscarinic (M3) acetylcholine receptor (M3R) in the stria vascularis (SV), the spiral ganglion (SGg) and the auditory nerve (AN) of the mouse (p14) cochlea. (**a–a”’**) In the SV, M3R labeling (a’) predominantly overlapped with the labeling of the inward rectifier-type potassium channel Kir4.1 (Kir4.1, A”), which is expressed in strial intermediate cells [3]. (**b–b”’**) M3R was immunolabeled in the cytoplasm of SGg cells (b’) that express Kir4.1 (b”) in their membranes [31]. (**c–c”’**) Strong M3R labeling was detected in the central part of the AN (VGg, vestibular ganglion). Immunolocalization of M3R in SV, SGg cells and AN is consistent with a previous report on M3R localization in the rat cochlea [51]. Scale bars: (a**–**b”’), 10 μm; (c**–**c”’), 100 μm. (JPEG 5225 kb)

Supplementary Figure 5 Immunolocalization of the muscarinic (M3) acetylcholine receptor (M3R) in secretory acinar cells of the salivary (parotid) gland from the p14 mouse. (**a–a”’**) In the parotid gland, immunolabeling of M3R (a’) overlapped with E-cadherin (a”)-labeled epithelial acinar cells (white arrowheads, a”’). (**b–b”’**) Higher magnification of parotid gland acinar cells reveals a partial co-localization of M3R (b’) with E-cadherin (b”) in their basolateral cell membranes (yellow spots, b”’). (**c–c”’**) Pre-incubation of the primary M3R antibody (0.1 μg/μL) with the corresponding immunizing peptide (0.2 μg/μL) resulted in a complete loss of M3R fluorescence (c’) in acinar cells. (**d–d”’**) No specific M3R (d’) or E-cadherin (d”) fluorescence signals were detected in acinar cells when both primary antibodies were omitted. * in (b”’), (c”’) and (d”’) indicates the acinar lumen; Scale bars: (a**–**a”’), 10 μm; (b**–**d”’), 5 μm. (JPEG 6908 kb)

Supplementary Figure 6 (**a–a”’**) Co-localization of AQP5 and the lipid raft-marker Flottilin-2 (Flot-2) in OSCs in the apical turn of a cochlear specimen from the ‘pilocarpine’ experimental group. Stripes of strong AQP5 (a’) and Flot-2 (a”) fluorescence are co-localized in the apical membranes (white arrowheads in (a”’)). Only weak AQP5 and Flot-2 fluorescence is present in the cytoplasmic regions (*, endolymphatic fluid space). This suggests the storage of AQP5 water channel proteins in lipid rafts that are translocated into the apical membrane of OSCs following M3R stimulation, which has previously been described for salivary gland acinar cells [40, 42]. (**b–c”’**) Representative confocal images of AQP5 (b’ and c’) and phalloidin fluorescence (b” and c”) in acinar cells of salivary (parotid) gland specimens from the ‘pilocarpine’ (b–b”’) and ‘pilocarpine + atropine’ (c–c”’) experimental groups. The inlays in (b’) and (c’) show the color-coded AQP5 fluorescence intensity (dark blue, low intensity; dark red, high intensity). Scale bars: (a–a”’), 5 μm; (b**–**c”’), 10 μm. (JPEG 4618 kb)

Supplementary Figure 7 Immunolocalization of AQP5 and M3R in acinar cells of the human salivary (parotid) gland. (**a–a”’**) AQP5 fluorescence is predominantly localized in the apical parts of acinar cells that border the acinar lumen with their apical membranes (white arrowheads, a”’). (**b–d”**) M3R is localized in the acinar cells of the human parotid gland (b–b”). Omitting the primary anti-M3R antibody (c–c”) or pre-incubation of the primary antibody (0.1 μg/μL) with the corresponding control peptide (0.2 μg/μL; d–d”) resulted in the complete absence of M3R fluorescence in acinar cells. (**e–e”’**) Immunolocalization of AQP4 in the murine kidney (inner medulla region; positive control). AQP4 fluorescence was detected exclusively in the inner medullary collecting duct (IMCD) epithelium. On the subcellular level, AQP4 was localized in the basolateral membranes of IMCD epithelial cells (inlay in e”’). Omitting the primary anti-AQP4 antibody resulted in the complete absence of AQP4 fluorescence signals in the IMCD epithelium (data not shown). Scale bars: (a–d”), 5 μm; (e–e”’), 50 μm; inlay in e”’, 5 μm. (JPEG 5749 kb)

Supplementary Table 1 Experimental groups from in vitro perilymphatic perfusion/incubation experiments. (pilocarpine, muscarinic M3 receptor (M3R) agonist; atropine, M3R antagonist. All substances were dissolved in HEPES–buffered Hank’s solution (HHBSS)). (PDF 30 kb)

Supplementary Table 2 Experimental groups from in vitro muscarinic M3 receptor fluorescent ligand-binding experiments. (M3-633-AN, muscarinic M3 receptor (M3R)-specific fluorescent ligand; 4-DAMP, unlabeled M3R competitor 4–diphenylacetoxy–N–methyl–piperidine methiodide; DMSO, dimethyl sulfoxide. All substances were dissolved in HEPES–buffered Hank’s solution (HHBSS)). (PDF 38 kb)

Supplementary Table 3 qRT-PCR data shown as ΔΔ CT values. (SL, spiral ligament; SV, stria vascularis; OC, organ of Corti; AN, auditory nerve with spiral ganglion; SG, salivary gland; SC, spinal cord; SI, small intestine; CI, confidence interval; n = number of experiments in triplicates). Data sets were tested for log–normal distribution, logarithmized, and tested for significance using Student’s t–test). (PDF 24 kb)

## References

[1] Adamzik M, Frey UH, Bitzer K, Jakob H, Baba HA, Schmieder RE, Schneider MP, Heusch G, Peters J, Siffert W (2008). A novel-1364A/C aquaporin 5 gene promoter polymorphism influences the responses to salt loading of the renin-angiotensin-aldosterone system and of blood pressure in young healthy men. Basic Res Cardiol.

[2] Andersen HC (1948). Passage of trypan blue into the endolymphatic system of the labyrinth. Acta Otolaryngol.

[3] Ando M, Takeuchi S (1999). Immunological identification of an inward rectifier K+ channel (Kir4.1) in the intermediate cell (melanocyte) of the cochlear stria vascularis of gerbils and rats. Cell Tissue Res.

[4] Angelborg C (1974). Distribution of macromolecular tracer particles (Thorotrast-r) in the cochlea. An electron microscopic study in guinea pig. Part I. The organ of Corti, the basilar membrane and the tympanic covering layer. Acta Otolaryngol Suppl.

[5] Angelborg C, Klockhoff I, Stahle J (1973). Serum osmolality in patients with Meniere’s disease. Acta Otolaryngol.

[6] Aoki M, Ando K, Kuze B, Mizuta K, Hayashi T, Ito Y (2005). The association of antidiuretic hormone levels with an attack of Meniere’s disease. Clin Otolaryngol.

[7] Bock GR, Steel KP (1983). Inner ear pathology in the deafness mutant mouse. Acta Otolaryngol.

[8] Bosher SK, Warren RL (1971). A study of the electrochemistry and osmotic relationships of the cochlear fluids in the neonatal rat at the time of the development of the endocochlear potential. J Physiol.

[9] Buijs RM, Swaab DF, Aminoff MJ, Boller F, Swaab DF (2013). Handbook of clinical neurology—autonomic nervous system.

[10] Cao X, Yin J, Wang H, Zhao J, Zhang J, Dai L, Zhang J, Jiang H, Lin Z, Yang Y (2014). Mutation in AQP5, encoding aquaporin 5, causes palmoplantar keratoderma Bothnia type. J Invest Dermatol.

[11] Celestino D, Iannetti G (1973). Meniere’s disease and plasmatic hyperosmolarity. J Laryngol Otol.

[12] Celestino D, Ralli G (1981). Plasmatic osmolality in Meniere’s disease. J Laryngol Otol.

[13] Chiba T, Marcus DC (2000). Nonselective cation and BK channels in apical membrane of outer sulcus epithelial cells. J Membr Biol.

[14] Chiba T, Marcus DC (2001). Basolateral K+ conductance establishes driving force for cation absorption by outer sulcus epithelial cells. J Membr Biol.

[15] Choi CH, Oghalai JS (2008). Perilymph osmolality modulates cochlear function. Laryngoscope.

[16] Conner AC, Bill RM, Conner MT (2013). An emerging consensus on aquaporin translocation as a regulatory mechanism. Mol Membr Biol.

[17] De Vincentiis I, Celestino D, Iannetti G (1972). New pathogenic and therapeutic conceptions in Meniere’s disease. Minerva Otorhinolaryngol.

[18] Duvall AJ, Santi PA, Hukee MJ (1980). Cochlear fluid balance. A clinical/research overview. Ann Otol Rhinol Laryngol.

[19] Eckhard A, Gleiser C, Arnold H, Rask-Andersen H, Kumagami H, Muller M, Hirt B, Lowenheim H (2012). Water channel proteins in the inner ear and their link to hearing impairment and deafness. Mol Aspects Med.

[20] Eckhard A, Gleiser C, Rask-Andersen H, Arnold H, Liu W, Mack A, Muller M, Lowenheim H, Hirt B (2012). Co-localisation of K(ir)4.1 and AQP4 in rat and human cochleae reveals a gap in water channel expression at the transduction sites of endocochlear K(+) recycling routes. Cell Tissue Res.

[21] Eckhard A, Muller M, Salt A, Smolders J, Rask-Andersen H, Lowenheim H (2014). Water permeability of the mammalian cochlea: functional features of an aquaporin-facilitated water shunt at the perilymph-endolymph barrier. Pflugers Arch.

[22] Giebel W (1982). The dynamic behavior of inner ear fluids. Laryngol Rhinol Otol (Stuttg).

[23] Gisselsson L (1949). The passage of fluorescein sodium to the labyrinthine fluids. Acta Otolaryngol.

[24] Gresz V, Horvath A, Gera I, Nielsen S, Zelles T (2014). Immunolocalization of AQP5 in resting and stimulated normal labial glands and in Sjogren’s syndrome. Oral Dis.

[25] Gresz V, Kwon TH, Gong H, Agre P, Steward MC, King LS, Nielsen S (2004). Immunolocalization of AQP-5 in rat parotid and submandibular salivary glands after stimulation or inhibition of secretion *in vivo*. Am J Physiol Gastrointest Liver Physiol.

[26] Griffin MT, Matsui M, Shehnaz D, Ansari KZ, Taketo MM, Manabe T, Ehlert FJ (2004). Muscarinic agonist-mediated heterologous desensitization in isolated ileum requires activation of both muscarinic M2 and M3 receptors. J Pharmacol Exp Ther.

[27] Guild SR (1927). The circulation of the endolymph. Am J Anat.

[28] Gulley RL, Reese TS (1976). Intercellular junctions in the reticular lamina of the organ of Corti. J Neurocytol.

[29] Haley GE, Flynn FW (2007). Tachykinin NK3 receptor contribution to systemic release of vasopressin and oxytocin in response to osmotic and hypotensive challenge. Am J Physiol Regul Integr Comp Physiol.

[30] Hasler U, Vinciguerra M, Vandewalle A, Martin PY, Feraille E (2005). Dual effects of hypertonicity on aquaporin-2 expression in cultured renal collecting duct principal cells. J Am Soc Nephrol.

[31] Hibino H, Horio Y, Fujita A, Inanobe A, Doi K, Gotow T, Uchiyama Y, Kubo T, Kurachi Y (1999). Expression of an inwardly rectifying K(+) channel, Kir4.1, in satellite cells of rat cochlear ganglia. Am J Physiol.

[32] Hirt B, Gleiser C, Eckhard A, Mack AF, Muller M, Wolburg H, Lowenheim H (2011). All functional aquaporin-4 isoforms are expressed in the rat cochlea and contribute to the formation of orthogonal arrays of particles. Neuroscience.

[33] Hirt B, Penkova ZH, Eckhard A, Liu W, Rask-Andersen H, Muller M, Lowenheim H (2010). The subcellular distribution of aquaporin 5 in the cochlea reveals a water shunt at the perilymph-endolymph barrier. Neuroscience.

[34] Hoffert JD, Leitch V, Agre P, King LS (2000). Hypertonic induction of aquaporin-5 expression through an ERK-dependent pathway. J Biol Chem.

[35] Horner KC, Cazals Y (2005). Stress hormones in Meniere’s disease and acoustic neuroma. Brain Res Bull.

[36] Huang D, Chen P, Chen S, Nagura M, Lim DJ, Lin X (2002). Expression patterns of aquaporins in the inner ear: evidence for concerted actions of multiple types of aquaporins to facilitate water transport in the cochlea. Hear Res.

[37] Inoue H, Uemura T (1988). Sluggishness of pupillary light contraction in patients with Meniere’s disease. Acta Otolaryngol.

[38] Inoue R, Sohara E, Rai T, Satoh T, Yokozeki H, Sasaki S, Uchida S (2013). Immunolocalization and translocation of aquaporin-5 water channel in sweat glands. J Dermatol Sci.

[39] Ishida N, Hirai SI, Mita S (1997). Immunolocalization of aquaporin homologs in mouse lacrimal glands. Biochem Biophys Res Commun.

[40] Ishikawa Y, Cho G, Yuan Z, Inoue N, Nakae Y (2006). Aquaporin-5 water channel in lipid rafts of rat parotid glands. Biochim Biophys Acta.

[41] Ishikawa Y, Eguchi T, Skowronski MT, Ishida H (1998). Acetylcholine acts on M3 muscarinic receptors and induces the translocation of aquaporin5 water channel via cytosolic Ca2+ elevation in rat parotid glands. Biochem Biophys Res Commun.

[42] Ishikawa Y, Yuan Z, Inoue N, Skowronski MT, Nakae Y, Shono M, Cho G, Yasui M, Agre P, Nielsen S (2005). Identification of AQP5 in lipid rafts and its translocation to apical membranes by activation of M3 mAChRs in interlobular ducts of rat parotid gland. Am J Physiol Cell Physiol.

[43] Jagger DJ, Nevill G, Forge A (2010). The membrane properties of cochlear root cells are consistent with roles in potassium recirculation and spatial buffering. J Assoc Res Otolaryngol.

[44] Jahnke K (1975). The fine structure of freeze-fractured intercellular junctions in the guinea pig inner ear. Acta Otolaryngol Suppl.

[45] Jahnke K (1980). Permeability barriers of the inner ear. Fine structure and function. Fortschr Med.

[46] Jefferis AF, Johnstone BM (1987). Plasma osmolality variations and their effect on the hearing threshold of the guinea pig. J Laryngol Otol.

[47] Juhn SK, Ikeda K, Morizono T, Murphy M (1991). Pathophysiology of inner ear fluid imbalance. Acta Otolaryngol Suppl.

[48] Juhn SK, Prado S (1976). The effect of hyperosmotic agents on perilymph osmolality. Trans Sect Otolaryngol Am Acad Ophthalmol Otolaryngol.

[49] Juhn SK, Prado S, Pearce J (1976). Osmolality changes in perilymph after systemic administration of glycerin. Arch Otolaryngol.

[50] Kakigi A, Takeda T (2009). Antidiuretic hormone and osmolality in patients with Meniere’s disease. ORL J Otorhinolaryngol Relat Spec.

[51] Khan KM, Drescher MJ, Hatfield JS, Khan AM, Drescher DG (2002). Muscarinic receptor subtypes are differentially distributed in the rat cochlea. Neuroscience.

[52] Kimura R, Schuknecht HF (1965). Membranous hydrops in the inner ear of the guinea pig after obliteration of the endolymphatic sac. Pract Otorhinolaryngol.

[53] Kitano H, Kitahara M (1987). Serum osmolality in Meniere’s disease. Am J Otol.

[54] Klockhoff I, Lindblom U (1966). Glycerol test in Meniere’s disease. Acta Otolaryngol.

[55] Lang F, Vallon V, Knipper M, Wangemann P (2007). Functional significance of channels and transporters expressed in the inner ear and kidney. Am J Physiol Cell Physiol.

[56] Lee BH, Gauna AE, Perez G, Park YJ, Pauley KM, Kawai T, Cha S (2013). Autoantibodies against muscarinic type 3 receptor in Sjogren’s syndrome inhibit aquaporin 5 trafficking. PLoS One.

[57] Lee JH, Chiba T, Marcus DC (2001). P2X2 receptor mediates stimulation of parasensory cation absorption by cochlear outer sulcus cells and vestibular transitional cells. J Neurosci.

[58] Lee JH, Marcus DC (2003). Endolymphatic sodium homeostasis by Reissner’s membrane. Neuroscience.

[59] Li J, Verkman AS (2001). Impaired hearing in mice lacking aquaporin-4 water channels. J Biol Chem.

[60] Livak KJ, Schmittgen TD (2001). Analysis of relative gene expression data using real-time quantitative PCR and the 2(-Delta Delta C(T)) Method. Methods.

[61] Manni JJ, Kuijpers W, van Wichem P (1986). Experimental endolymphatic hydrops in the rat. Arch Otolaryngol Head Neck Surg.

[62] Marcus DC, Chiba T (1999). K+ and Na + absorption by outer sulcus epithelial cells. Hear Res.

[63] Masuyama K, Uno K, Minoda R, Eura M, Samejima Y, Ishikawa T (1996). Muscarinic acetylcholine receptors on human lymphocytes in patients with Meniere’s disease. Acta Otolaryngol.

[64] Mikaelian D, Ruben RJ (1965). Development of hearing in the normal Cba-J Mouse: correlation of physiological observations with behavioral responses and with cochlear anatomy. Acta Otolaryngol.

[65] Morrison GA, Teixeira M, Sterkers O, Amiel C, Ferrary E (1996). Effect of glycerol on electrochemical composition of endolymph and perilymph in the rat. Acta Otolaryngol.

[66] Murdiastuti K, Purwanti N, Karabasil MR, Li X, Yao C, Akamatsu T, Kanamori N, Hosoi K (2006). A naturally occurring point mutation in the rat aquaporin 5 gene, influencing its protein production by and secretion of water from salivary glands. Am J Physiol Gastrointest Liver Physiol.

[67] Naftalin L, Harrison MS (1958). Circulation of labyrinthine fluids. J Laryngol Otol.

[68] Nejsum LN, Kwon TH, Jensen UB, Fumagalli O, Frokiaer J, Krane CM, Menon AG, King LS, Agre PC, Nielsen S (2002). Functional requirement of aquaporin-5 in plasma membranes of sweat glands. Proc Natl Acad Sci U S A.

[69] Nishio N, Teranishi M, Uchida Y, Sugiura S, Ando F, Shimokata H, Sone M, Otake H, Kato K, Yoshida T, Tagaya M, Hibi T, Nakashima T (2013). Polymorphisms in genes encoding aquaporins 4 and 5 and estrogen receptor alpha in patients with Meniere’s disease and sudden sensorineural hearing loss. Life Sci.

[70] Orji F (2014). The influence of psychological factors in Meniere’s disease. Ann Med Health Sci Res.

[71] Pogson ZE, McKeever TM, Fogarty A (2008). The association between serum osmolality and lung function among adults. Eur Respir J.

[72] Rudert H (1969). Investigation on resorption of the endolymph in the inner ear of the guinea pig. 3. Electron microscope investigations on the structure of the endolymphatic sac and ferritin resorption in the membranous labyrinth. Arch Klin Exp Ohren Nasen Kehlkopfheilkd.

[73] Rudert H (1969). Investigation on resorption of the endolymph in the inner ear of the guinea pig. I. Microscopic examinations after injection of trypan blue into the cochlear duct. Arch Klin Exp Ohren Nasen Kehlkopfheilkd.

[74] Rudert H (1969). Investigation on resorption of the endolymph in the inner ear of the guinea pig. II. Experiments with radioactive labeled substances and autoradiographic evaluation. Arch Klin Exp Ohren Nasen Kehlkopfheilkd.

[75] Salt AN, DeMott J (1997). Longitudinal endolymph flow associated with acute volume increase in the guinea pig cochlea. Hear Res.

[76] Salt AN, DeMott JE (1995). Endolymph volume changes during osmotic dehydration measured by two marker techniques. Hear Res.

[77] Smith CA, Lowry OH, Wu ML (1954). The electrolytes of the labyrinthine fluids. Laryngoscope.

[78] Song Y, Sonawane N, Verkman AS (2002). Localization of aquaporin-5 in sweat glands and functional analysis using knockout mice. J Physiol.

[79] Sterkers O, Ferrary E, Amiel C (1984). Inter- and intracompartmental osmotic gradients within the rat cochlea. Am J Physiol.

[80] Sterkers O, Saumon G, Tran Ba Huy P, Amiel C (1982). K, Cl, and H2O entry in endolymph, perilymph, and cerebrospinal fluid of the rat. Am J Physiol.

[81] Uchide K, Suzuki N, Takiguchi T, Terada S, Inoue M (1997). The possible effect of pregnancy on Meniere’s disease. ORL J Otorhinolaryngol Relat Spec.

[82] Ueda H, Muratsuka Y, Konishi T (1987). Effect of glycerol on inner ear fluid electrolytes and osmolalities in guinea pigs. Ann Otol Rhinol Laryngol.

[83] Uemura T, Inoue H, Matsunaga K (1985). Pupillary dynamics in patients with Meniere’s disease. Am J Otolaryngol.

[84] Uemura T, Itoh M, Kikuchi N (1980). Autonomic dysfunction on the affected side in Meniere’s disease. Acta Otolaryngol.

[85] van Cruijsen N, Dullaart RP, Wit HP, Albers FW (2005). Analysis of cortisol and other stress-related hormones in patients with Meniere’s disease. Otol Neurotol.

[86] Wangemann P (2002). Adrenergic and muscarinic control of cochlear endolymph production. Adv Otorhinolaryngol.

[87] Wangemann P, Liu J, Scherer EQ, Herzog M, Shimozono M, Scofield MA (2001). Muscarinic receptors control K+ secretion in inner ear strial marginal cells. J Membr Biol.

[88] Woo J, Chae YK, Jang SJ, Kim MS, Baek JH, Park JC, Trink B, Ratovitski E, Lee T, Park B, Park M, Kang JH, Soria JC, Lee J, Califano J, Sidransky D, Moon C (2008). Membrane trafficking of AQP5 and cAMP dependent phosphorylation in bronchial epithelium. Biochem Biophys Res Commun.

[89] Yang F, Kawedia JD, Menon AG (2003). Cyclic AMP regulates aquaporin 5 expression at both transcriptional and post-transcriptional levels through a protein kinase A pathway. J Biol Chem.

[90] Yildiz SK, Koybasi S, Turkoglu SA, Yildiz N, Korkmaz B, Akyurek F (2007). Sympathetic skin responses from postauricular region in Meniere’s disease. Clin Neurophysiol.

[91] Zidanic M, Brownell WE (1990). Fine structure of the intracochlear potential field. I. The silent current. Biophys J.

