# Supplementary figures and images for: Regulation of the perilymphatic–endolymphatic water shunt in the cochlea by membrane translocation of aquaporin-5
Source: Pflugers Arch. 2015 Jul 25;467(12):2571–88. doi: 10.1007/s00424-015-1720-6 (PMC4646919; doi:10.1007/s00424-015-1720-6)

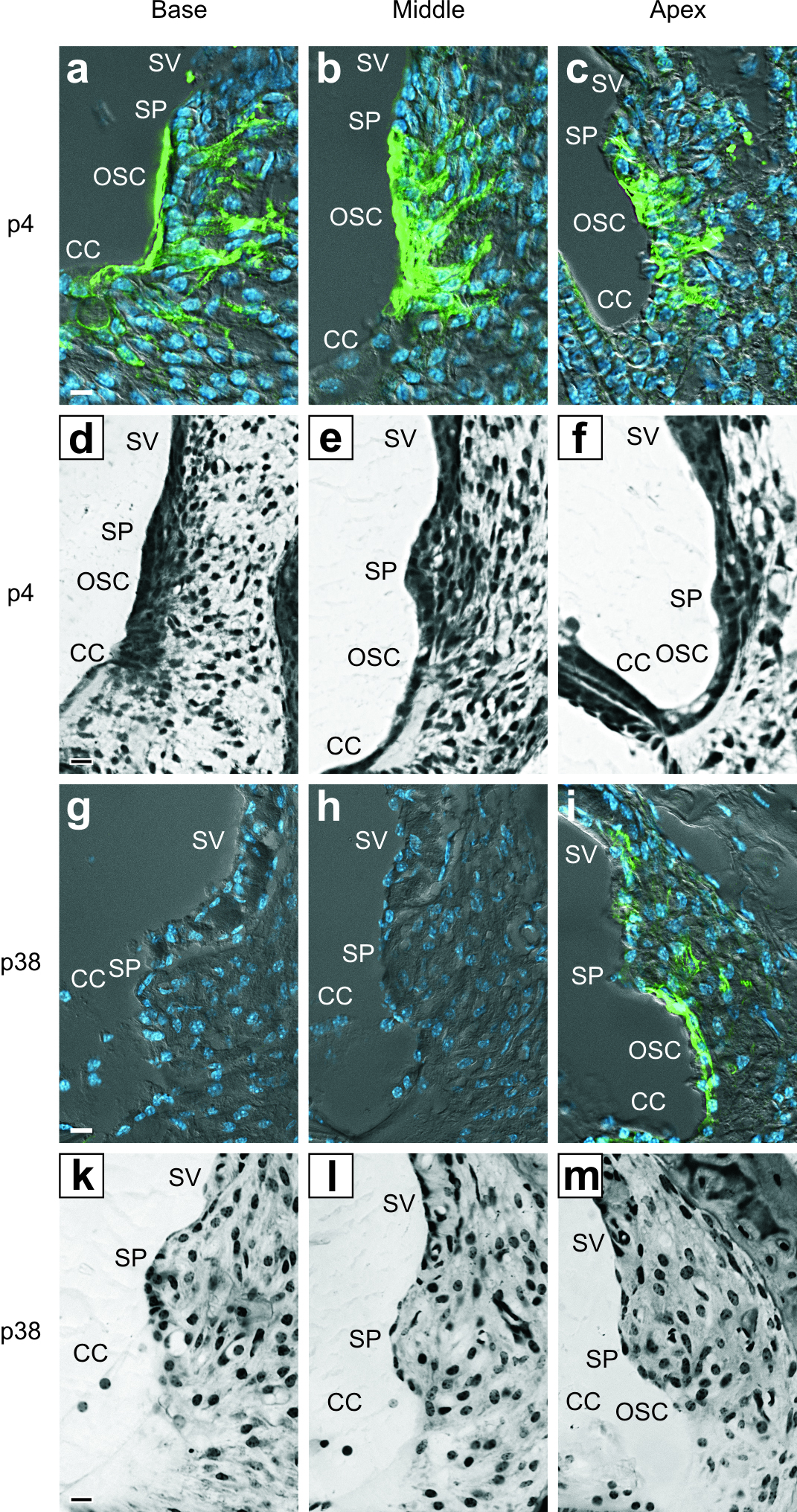

Supplement: Supplementary file 1 — Morphology of the outer sulcus region and AQP5 expression in outer sulcus cells (OSCs) in the apex and the base of the early postnatal (p4) and mature (p38) murine cochlea. (a–c) At p4, AQP5 (green) is expressed in OSCs in the base (a), middle (b), and apex (c) of the cochlea (DAPI, blue) (also see Fig. 1a and b). (d–f) Epon sections of the p4 cochlea demonstrate that OSCs in all three turns are between the Claudius cells (CC) and spiral prominence (SP) epithelial cells and that OSCs in all turns contact the endolymphatic fluid space with their apical membranes. (g–i) At p38, AQP5 expression (green) disappears in OSCs in the basal (g) and middle turn (h) and is restricted to OSCs in the cochlear apex (i; DAPI, blue) (also see Fig. 1a and b). (k–m) Epon sections of the p38 cochlea reveal that OSCs in the basal and middle turn were overgrown by CCs and therefore lost physical contact with the endolymphatic fluid space. In the outer sulcus region in the cochlear apex, the early postnatal morphological configuration is preserved, with AQP5-expressing OSCs between the CCs and SP epithelial cells. Thus, this subpopulation of OSCs retains its direct contact to the endolymphatic fluid space. (SV, stria vascularis). Scale bars: 20 μm. (JPEG 2888 kb) [file 424_2015_1720_MOESM1_ESM.jpg]

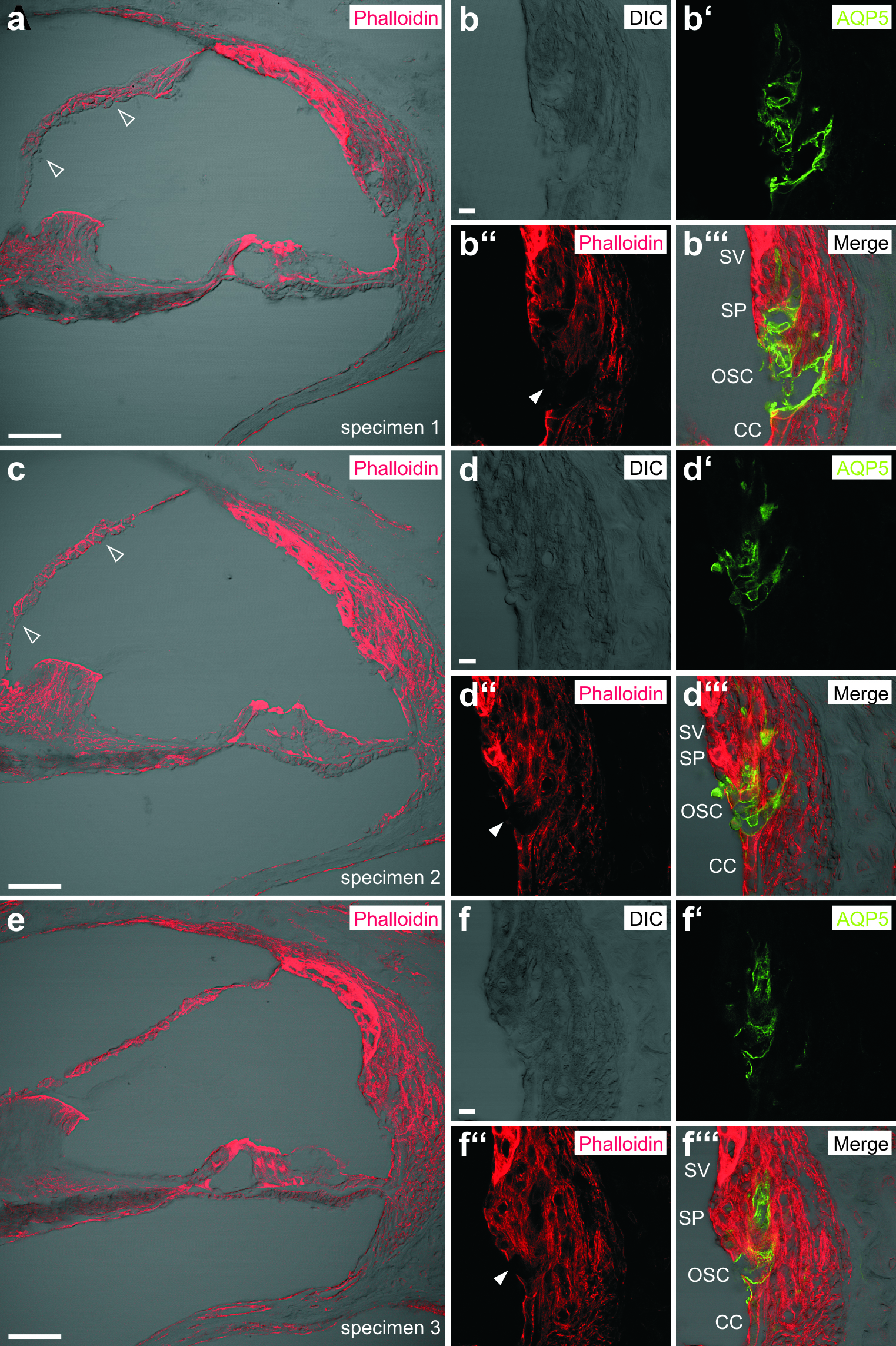

Supplement: Supplementary file 2 — Cellular damage in OSCs in the apical cochlear turn induced by perilymphatic hypoosmolarity. (a, c and e) Overview of the cochlear duct in the apical turn from the three specimens in the ‘hypoosmolar’ group. Bulging of Reissner’s membrane into scala vestibuli was observed in specimens 1 and 2 (hollow arrowheads, (a) and (c)), which suggests osmotic-driven water flow into the endolymph fluid space. (b–f”’) Higher magnification of the outer sulcus area from specimens 1 (b–b”’), 2 (d–d”’), and 3 (f–f”’). Apical membranes of OSCs are disrupted as indicated by the loss of phalloidin fluorescence at the luminal membrane borders (white arrowheads in (b”), (d”) and (f”)). The neighboring spiral prominence (SP) epithelial cells and Claudius cells (CC) show intact cellular outlines based on their membranous phalloidin fluorescence. Scale bars: (a, c, e), 100 μm; (b–b”’, d–d”’, f–f”’), 10 μm. (JPEG 4370 kb) [file 424_2015_1720_MOESM2_ESM.jpg]

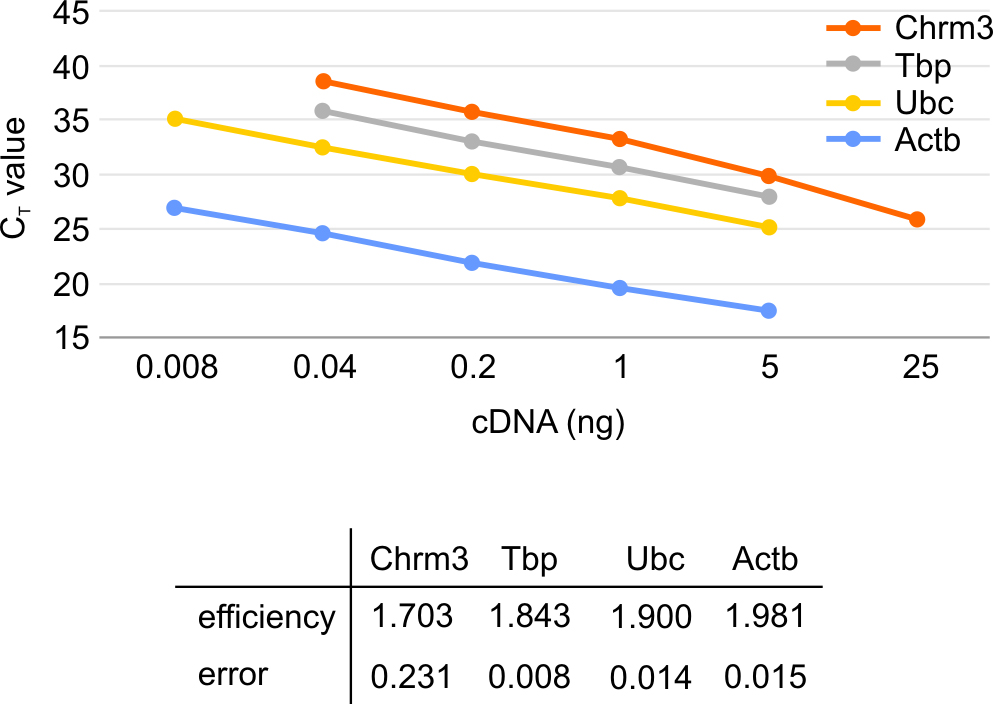

Supplement: Supplementary file 3 — Primer efficiencies and errors were determined using standard curve experiments on murine (p4) organ of Corti cDNA (for reference genes) or murine (p14) spinal cord cDNA (for Chrm3) in a dose-dependent fashion (8 pg–25 ng of cDNA per reaction). The CT value corresponds to the cycle of amplification in which the fluorescence of a sample surpassed the background fluorescence, and the CT values were determined using the LightCycler® 480 Software release 1.5.0 SP4 (Roche Diagnostics). Mean CT value ± SD (triplicates). (JPEG 211 kb) [file 424_2015_1720_MOESM3_ESM.jpg]

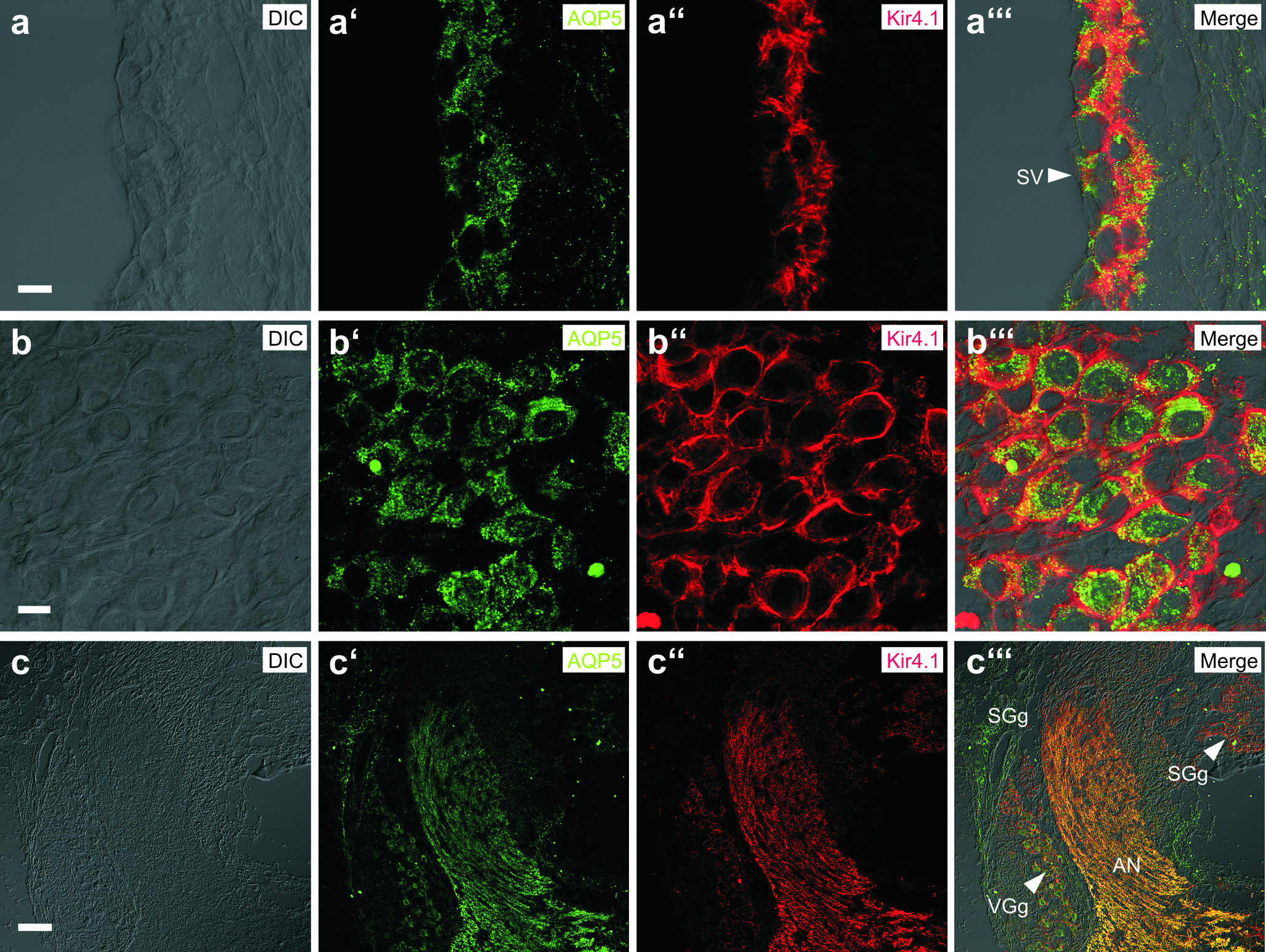

Supplement: Supplementary file 4 — Immunolocalization of the muscarinic (M3) acetylcholine receptor (M3R) in the stria vascularis (SV), the spiral ganglion (SGg) and the auditory nerve (AN) of the mouse (p14) cochlea. (a–a”’) In the SV, M3R labeling (a’) predominantly overlapped with the labeling of the inward rectifier-type potassium channel Kir4.1 (Kir4.1, A”), which is expressed in strial intermediate cells [3]. (b–b”’) M3R was immunolabeled in the cytoplasm of SGg cells (b’) that express Kir4.1 (b”) in their membranes [31]. (c–c”’) Strong M3R labeling was detected in the central part of the AN (VGg, vestibular ganglion). Immunolocalization of M3R in SV, SGg cells and AN is consistent with a previous report on M3R localization in the rat cochlea [51]. Scale bars: (a–b”’), 10 μm; (c–c”’), 100 μm. (JPEG 5225 kb) [file 424_2015_1720_MOESM4_ESM.jpg]

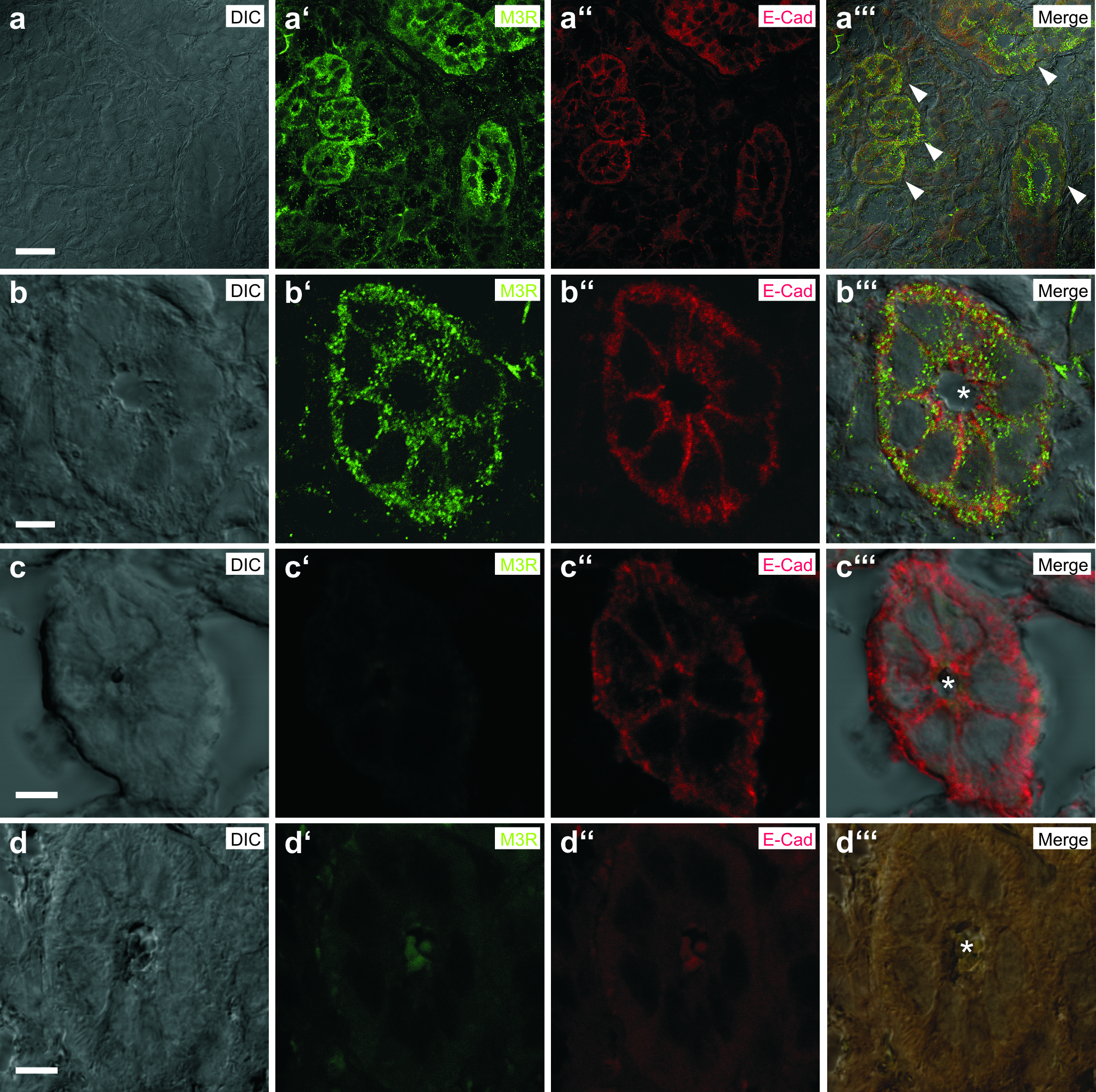

Supplement: Supplementary file 5 — Immunolocalization of the muscarinic (M3) acetylcholine receptor (M3R) in secretory acinar cells of the salivary (parotid) gland from the p14 mouse. (a–a”’) In the parotid gland, immunolabeling of M3R (a’) overlapped with E-cadherin (a”)-labeled epithelial acinar cells (white arrowheads, a”’). (b–b”’) Higher magnification of parotid gland acinar cells reveals a partial co-localization of M3R (b’) with E-cadherin (b”) in their basolateral cell membranes (yellow spots, b”’). (c–c”’) Pre-incubation of the primary M3R antibody (0.1 μg/μL) with the corresponding immunizing peptide (0.2 μg/μL) resulted in a complete loss of M3R fluorescence (c’) in acinar cells. (d–d”’) No specific M3R (d’) or E-cadherin (d”) fluorescence signals were detected in acinar cells when both primary antibodies were omitted. * in (b”’), (c”’) and (d”’) indicates the acinar lumen; Scale bars: (a–a”’), 10 μm; (b–d”’), 5 μm. (JPEG 6908 kb) [file 424_2015_1720_MOESM5_ESM.jpg]

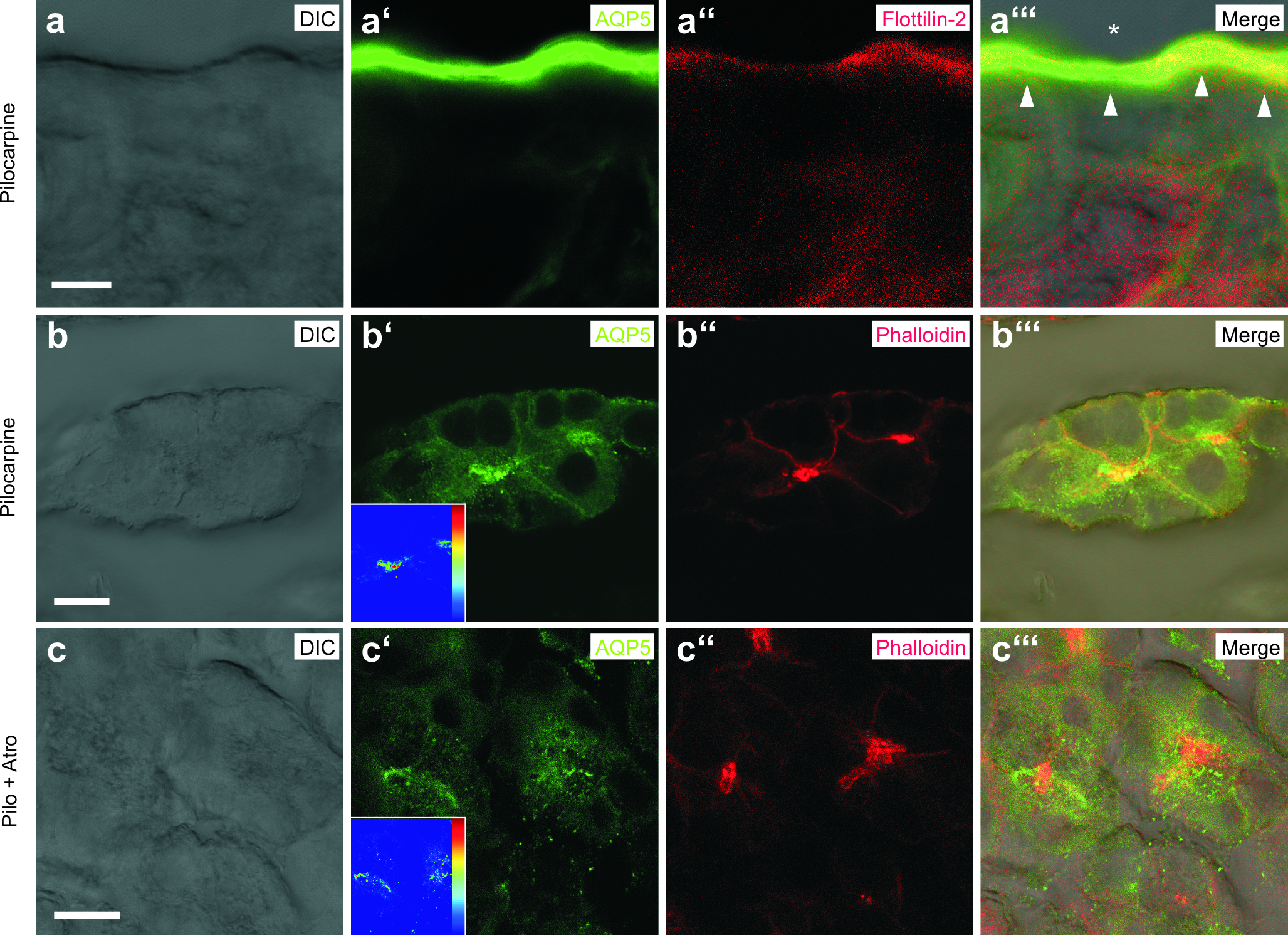

Supplement: Supplementary file 6 — (a–a”’) Co-localization of AQP5 and the lipid raft-marker Flottilin-2 (Flot-2) in OSCs in the apical turn of a cochlear specimen from the ‘pilocarpine’ experimental group. Stripes of strong AQP5 (a’) and Flot-2 (a”) fluorescence are co-localized in the apical membranes (white arrowheads in (a”’)). Only weak AQP5 and Flot-2 fluorescence is present in the cytoplasmic regions (*, endolymphatic fluid space). This suggests the storage of AQP5 water channel proteins in lipid rafts that are translocated into the apical membrane of OSCs following M3R stimulation, which has previously been described for salivary gland acinar cells [40, 42]. (b–c”’) Representative confocal images of AQP5 (b’ and c’) and phalloidin fluorescence (b” and c”) in acinar cells of salivary (parotid) gland specimens from the ‘pilocarpine’ (b–b”’) and ‘pilocarpine + atropine’ (c–c”’) experimental groups. The inlays in (b’) and (c’) show the color-coded AQP5 fluorescence intensity (dark blue, low intensity; dark red, high intensity). Scale bars: (a–a”’), 5 μm; (b–c”’), 10 μm. (JPEG 4618 kb) [file 424_2015_1720_MOESM6_ESM.jpg]

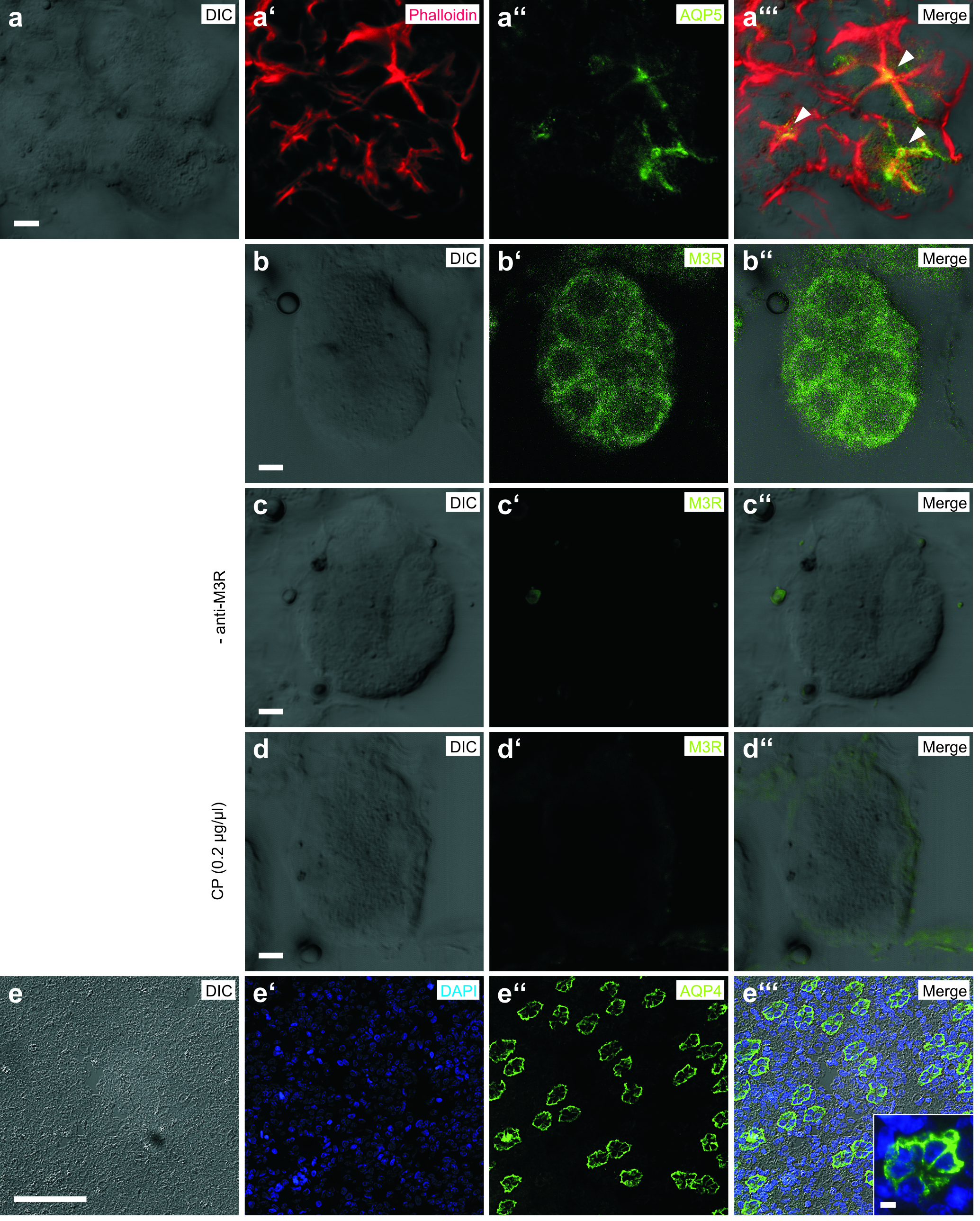

Supplement: Supplementary file 7 — Immunolocalization of AQP5 and M3R in acinar cells of the human salivary (parotid) gland. (a–a”’) AQP5 fluorescence is predominantly localized in the apical parts of acinar cells that border the acinar lumen with their apical membranes (white arrowheads, a”’). (b–d”) M3R is localized in the acinar cells of the human parotid gland (b–b”). Omitting the primary anti-M3R antibody (c–c”) or pre-incubation of the primary antibody (0.1 μg/μL) with the corresponding control peptide (0.2 μg/μL; d–d”) resulted in the complete absence of M3R fluorescence in acinar cells. (e–e”’) Immunolocalization of AQP4 in the murine kidney (inner medulla region; positive control). AQP4 fluorescence was detected exclusively in the inner medullary collecting duct (IMCD) epithelium. On the subcellular level, AQP4 was localized in the basolateral membranes of IMCD epithelial cells (inlay in e”’). Omitting the primary anti-AQP4 antibody resulted in the complete absence of AQP4 fluorescence signals in the IMCD epithelium (data not shown). Scale bars: (a–d”), 5 μm; (e–e”’), 50 μm; inlay in e”’, 5 μm. (JPEG 5749 kb) [file 424_2015_1720_MOESM7_ESM.jpg]
