# Supplementary material for: Regulation of the perilymphatic–endolymphatic water shunt in the cochlea by membrane translocation of aquaporin-5
Source: Pflugers Arch. 2015 Jul 25;467(12):2571–88. doi: 10.1007/s00424-015-1720-6 (PMC4646919; doi:10.1007/s00424-015-1720-6)
Supplement: Supplementary file 8 — Experimental groups from in vitro perilymphatic perfusion/incubation experiments. (pilocarpine, muscarinic M3 receptor (M3R) agonist; atropine, M3R antagonist. All substances were dissolved in HEPES–buffered Hank’s solution (HHBSS)). (PDF 30 kb) [file 424_2015_1720_MOESM8_ESM.pdf]

Experimental group name	Perilymphatic perfusate osmolarity	M3 agonist/antagonist (conc.)	Number of cochlear specimens analyzed (n)
hyposomolarity	200 mOsm/L	-	3
hyperosmolarity	400 mOsm/L	-	3
isoosmolarity (control)	285 mOsm/L	-	3
pilocarpine	285 mOsm/L	Pilocarpine (10 µM)	3
pilocarpine + atropine	285 mOsm/L	Pilocarpine (10 µM)<br>Atropine (100 µM)	3
hyperosmolarity + atropine	400 mOsm/L	Atropine (100 µM)	3
