# Supplementary material for: Regulation of the perilymphatic–endolymphatic water shunt in the cochlea by membrane translocation of aquaporin-5
Source: Pflugers Arch. 2015 Jul 25;467(12):2571–88. doi: 10.1007/s00424-015-1720-6 (PMC4646919; doi:10.1007/s00424-015-1720-6)
Supplement: Supplementary file 9 — Experimental groups from in vitro muscarinic M3 receptor fluorescent ligand-binding experiments. (M3-633-AN, muscarinic M3 receptor (M3R)-specific fluorescent ligand; 4-DAMP, unlabeled M3R competitor 4–diphenylacetoxy–N–methyl–piperidine methiodide; DMSO, dimethyl sulfoxide. All substances were dissolved in HEPES–buffered Hank’s solution (HHBSS)). (PDF 38 kb) [file 424_2015_1720_MOESM9_ESM.pdf]

**Experimental group name**	**Pre-incubation media (conc., time)**	**Incubation media (conc., time)**	**Number of cochlear specimens analyzed (n)**
M3-633-AN	DMSO (0.1%, 10 min)	M3-633-AN in 0.1% DMSO (100nM, 20 min)	3
4-DAMP	4-DAMP (10 μM, 10 min)	M3-633-AN in 0.1% DMSO (100nM, 20 min)	3
DMSO (control)	DMSO (0.1%, 10 min)	DMSO (0.1%, 20 min)	3
