# Supplementary material for: Regulation of the perilymphatic–endolymphatic water shunt in the cochlea by membrane translocation of aquaporin-5
Source: Pflugers Arch. 2015 Jul 25;467(12):2571–88. doi: 10.1007/s00424-015-1720-6 (PMC4646919; doi:10.1007/s00424-015-1720-6)
Supplement: Supplementary file 10 — qRT-PCR data shown as ΔΔ CT values. (SL, spiral ligament; SV, stria vascularis; OC, organ of Corti; AN, auditory nerve with spiral ganglion; SG, salivary gland; SC, spinal cord; SI, small intestine; CI, confidence interval; n = number of experiments in triplicates). Data sets were tested for log–normal distribution, logarithmized, and tested for significance using Student’s t–test). (PDF 24 kb) [file 424_2015_1720_MOESM10_ESM.pdf]

Target	Tissue sample	Mean	95% CI low	95% CI high	n
Chrm3					
	SL	1,1919	0,77	1,618	4
	SV	0,683	0,38	0,981	4
	OC	8,0517	4,78	11,323	4
	AN	21,4187	2,06	40,778	4
	SG	5,9928	2,06	9,927	3
	SC	38,8722	19,29	58,456	1
	SI	6,0927	-0,74	12,926	1

Target	Efficiency	Error	Comparison	Difference	95% CI low	95% CI high	p-Value	significant
Chrm3	2,31E-001	1,703						
			SL/AN	20,22681	8,8382	31,61542	<.0001	Yes
			SL/OC	6,85983	-4,5288	18,24843	<.0001	Yes
			SL/SC	37,68024	19,6733	55,68721	<.0001	Yes
			SL/SG	4,80087	-7,5002	17,10197	0,0046	Yes
			SL/SI	4,90079	-13,1062	22,90776	0,0076	Yes
			SL/SV	0,50891	-10,8797	11,89752	0,0857	No

Reference	Efficiency	Error
Tpp	1,843	0,008
Ubc	1,900	0,014
Actb	1,981	0,015
